# Systematic review of the methods of health economic models assessing antipsychotic medication for schizophrenia

**DOI:** 10.1371/journal.pone.0234996

**Published:** 2020-07-10

**Authors:** Huajie Jin, Paul Tappenden, Stewart Robinson, Evanthia Achilla, David Aceituno, Sarah Byford

**Affiliations:** 1 King’s Health Economics (KHE), Institute of Psychiatry, Psychology & Neuroscience at King’s College London, London, United Kingdom; 2 Health Economics and Decision Science, School of Health and Related Research, University of Sheffield, Sheffield, United Kingdom; 3 School of Business and Economics, Loughborough University, Loughborough, United Kingdom; Universitat Witten/Herdecke, GERMANY

## Abstract

**Background:**

Numerous economic models have assessed the cost-effectiveness of antipsychotic medications in schizophrenia. It is important to understand what key impacts of antipsychotic medications were considered in the existing models and limitations of existing models in order to inform the development of future models.

**Objectives:**

This systematic review aims to identify which clinical benefits, clinical harms, costs and cost savings of antipsychotic medication have been considered by existing models, to assess quality of existing models and to suggest good practice recommendations for future economic models of antipsychotic medications.

**Methods:**

An electronic search was performed on multiple databases (MEDLINE, EMBASE, PsycInfo, Cochrane database of systematic reviews, The NHS Economic Evaluation Database and Health Technology Assessment database) to identify economic models of schizophrenia published between 2005–2020. Two independent reviewers selected studies for inclusion. Study quality was assessed using the National Institute for Health and Care Excellence (NICE) checklist and the Cooper hierarchy. Key impacts of antipsychotic medications considered by exiting models were descriptively summarised.

**Results:**

Sixty models were included. Existing models varied greatly in key impacts of antipsychotic medication included in the model, especially in clinical outcomes used for assessing reduction in psychotic symptoms and types of adverse events considered in the model. Quality of existing models was generally low due to failure to capture the health and cost impact of adverse events of antipsychotic medications and input data not obtained from best available source. Good practices for modelling antipsychotic medications are suggested.

**Discussions:**

This review highlights inconsistency in key impacts considered by different models, and limitations of the existing models. Recommendations on future research are provided.

## Introduction

Antipsychotic medications have been the mainstay of treatment for schizophrenia since the 1950s [1]. They can be used for treating acute psychotic states in the short-term, or preventing relapse in the long-term, either as oral preparations or in the form of long-acting injectable (LAI) preparations. There are around twenty different antipsychotic medications licensed in Australia, Canada, the UK and the US [2–5], each of which is associated with different treatment efficacy and adverse event profiles [6]. Common adverse events of antipsychotic medications include extrapyramidal symptoms (EPS, such as parkinsonism, acute dystonic reactions, akathisia and tardive dyskinesia), increased prolactin levels, seizures, sedation, weight gain, diabetes and autonomic effects (such as blurring of vision, increased intra-ocular pressure, dry mouth and eyes, constipation and urinary retention) [7].

Clinical trials capturing the health and cost outcomes of antipsychotic medications can be complex, time-consuming, and costly. In addition, clinical trials often do not cover all antipsychotic medications of interest, have a short time horizon and use surrogate endpoints or focus only on specific adverse events instead of assessing the entire spectrum of relevant adverse events. Health economic modelling is therefore advocated as a systematic and transparent method for synthesising all available evidence obtained from multiple sources [8–11]. Although the results of economic models do not provide definitive answers as to how resources should be allocated, they act as a tool for use in the decision-making process by identifying what might happen when resources are allocated in different ways.

Models are simplifications of reality which focus only on key relationships and are therefore unable to capture all impacts of using one intervention [12]. Therefore, modellers need to determine the amount of detail to include in the model, including (i) clinical benefits (e.g. symptom relief and life extension), (ii) clinical harms (e.g. adverse events and complications), (iii) costs (e.g. cost of providing the intervention) and (iv) cost savings (e.g. cost of prevented relapses), as a consequence of using alternative interventions. If important impacts of an intervention are omitted, the model results will be flawed and resource allocation decisions may be incorrect [13], with potential consequences including wasted resources in the healthcare system and quality of life and survival losses in the population concerned. Several systematic reviews [7, 14–18] have been conducted for economic evaluations of antipsychotic medications for people with schizophrenia, and they all reported inconsistency in conclusions reported by different models. However, none of the published systematic reviews explored and compared the key clinical and cost impacts of antipsychotic medications considered by existing models. To fill this gap, this systematic review aims to identify which clinical benefits, clinical harms, costs and cost savings of antipsychotic medication have been considered by existing models, to assess quality of existing models and to suggest good practice recommendations for future economic models of antipsychotic medications.

## Methods

This systematic review was conducted according to the Preferred Reporting Items for Systematic Reviews and Meta-Analyses (PRISMA) recommendations for reporting systematic reviews and meta-analyses of studies that evaluate healthcare interventions [19]. The systematic review protocol is reported in S1 Table.

### Inclusion/exclusion criteria

Inclusion and exclusion criteria were defined *a priori*. Studies were included if they met all of the following criteria: (i) model-based economic evaluation adopting either a cost-effectiveness analysis (CEA) or cost-utility analysis (CUA) approach; (ii) young people (under 18 years of age) and/or adults (18 years and older) with a non-specific diagnosis of psychosis or with a diagnosis of schizophrenia (including schizoaffective disorder and delusional disorder); (iii) evaluation of antipsychotic medications versus each other, placebo or nothing. The reason why non-specific diagnosis of psychosis was included is because in clinical practice, it may take up many years before symptomatic patients receive a formal diagnosis of schizophrenia [20]. Before a formal diagnosis of schizophrenia can be made, patients with symptoms of schizophrenia often receive a less specific umbrella diagnosis of ‘psychosis’ [21]. No restrictions by country, setting or currency were applied. Studies were excluded if they met any of the following criteria: (i) reviews, commentaries, letters, editorials, or abstracts; (ii) published before 2005; and (iii) not reported in English. Although no language restrictions were applied to the search, only papers published in English language were included in the review.

### Search strategy

Electronic biomedical and psychological databases searched included MEDLINE (including in-Process & other non-indexed), EMBASE and PsycINFO, accessed through the Ovid interface (https://ovidsp.ovid.com/). In addition, the NHS Economic Evaluation Database (NHS EED) and the Health Technology Assessment (HTA) Database were searched, accessed through the Cochrane library interface (http://onlinelibrary.wiley.com/cochranelibrary/search8). The search strategies included Medical Subject Heading (MeSH) terms and text words. Each follows a similar structure: population terms AND economic evaluation terms AND modelling terms AND limitation terms. The original search, first update search and second update search were conducted by one reviewer (HJ) on 22nd June 2015, 4th March 2018, and 21st January 2020, respectively. The full search strategy is reported in S1 Text. Retrieved search results were downloaded into Endnote X8.0.2.

### Assessment of abstracts for inclusion

Two reviewers (HJ and EA for the original search and first update search, HJ and DA for the second update search) interpedently performed the first screening of the literature search results, by comparing titles and abstracts to the inclusion criteria. The full articles were then obtained for possibly useful studies and checked against the review inclusion criteria by two reviewers (HJ and EA for the studies retrieved from the original search and first update search, HJ and DA for the studies retrieved from the second update search). Final inclusion of studies in the review was determined by agreement of both reviewers, with disagreements resolved by discussion. A number of strategies were devised to help ensure that relevant studies were not missed. Firstly, the reference lists of all studies included in the current review were checked for any additional studies that may have been missed by the electronic search strategies. Secondly, key papers and the publications of key health economists were checked for inclusion and for additional relevant papers. Thirdly, published systematic reviews relevant to the target population were located through a separate search of the clinical guidelines and technology appraisals produced by the National Institute for Health and Care Excellence (NICE) and NIHR health technology assessment (HTA) reports. The search terms used by the located systematic reviews were used to inform the development of search strategies for the current systematic review, and the studies included by those reviews were checked for relevance to the inclusion criteria of the current systematic review. Finally, the included studies of all literature reviews identified by the search conducted for the current systematic review were checked for any additional studies that may have been missed by the electronic search strategies.

### Quality assessment

Seven commonly used checklists for economic evaluations [22–28] were considered for the current review, they differ from each other in terms of the aim of the quality assessment (e.g. to assess reporting quality, or methodological quality of economic evaluations, or both) and the types of studies covered (e.g. trial-based economic evaluations, model-based economic evaluations, or both). To be of value to the current review, checklists needed to (i) focus on methodological quality of studies; (ii) be appropriate for modelling studies; and (iii) provides an overall judgement regarding the methodological quality of the studies assessed, so to help the reviewers to summarise and compare the methodological quality of a large number of included studies (e.g. ≥50 studies). Based on these three criteria, two checklists were deemed to be most appropriate for the current review: Section 2 of the NICE checklist [22] and the Cooper hierarchy [23]. The NICE checklist consists of two sections. Section 1 aims to assess the applicability of a study to the decision problems that need to be addressed by NICE guidance. For example, is the system in which the study was conducted sufficiently similar to the current UK context? Is the study population appropriate for the review question of NICE guidance? As the aim of this systematic review was to explore the methods employed by existing models rather than to apply their results to the UK setting, Section 1 was not considered relevant for the purpose of this systematic review. Section 2 of the NICE checklist aims to assess the methodological quality of the study and thus was included. Section 2 consists of twelve quality criteria and an overall assessment. The quality criteria of Section 2 covered issues such as model structure, time horizon, source of input data and sensitivity analysis. Based on the number and importance of quality criteria that a study fails, an assessment regarding the overall methodological quality of the study can be classified into one of the following categories: (i) very serious limitations—the study fails to meet one or more quality criteria, and this is highly likely to change the conclusions about cost effectiveness, (ii) potentially serious limitations—the study fails to meet one or more quality criteria, and this could change the conclusions about cost effectiveness, and (iii) minor limitations—the study meets all quality criteria, or fails to meet one or more quality criteria but this is unlikely to change the conclusions about cost effectiveness, potentially serious limitations and minor limitations.

The Cooper hierarchy focuses only on the quality of the data sources used to inform the parameters in a model and was modified from the potential hierarchies of data sources for economic analyses developed by Coyle and Lee [29]. The Cooper hierarchy provides a list of potential sources for each data component of interest, including main clinical effect size, baseline clinical data, adverse events and complications, resource use, costs and utilities. Sources are ranked on a scale from 1 to 6, with the most appropriate source assigned a rank of 1. Where multiple data inputs were included within a category (i.e. adverse events and complications, resource use and cost), the score of the worst sources of evidence were recorded. Based on the value of the score, the quality of input data was then categorised as high ranked evidence (score 1–2), medium ranked evidence (score 3–4) or low ranked evidence (score 5–6). According to the Cooper hierarchy, the best source for clinical treatment effects and adverse events are meta-analyses of RCTs with direct comparison between comparator therapies, measuring final outcomes. The best sources for baseline clinical data are case series or analyses of reliable administrative databases specifically conducted for the study covering patients solely from the jurisdiction of interest. The best sources for resource use data are prospective data collection or analyses of reliable administrative data for the specific study. The best sources for unit cost data are cost calculations based on reliable databases or data sources created for the specific study (same jurisdiction). The best sources for utility data are direct utility assessment for the specific study from a sample (a) of the general population; (b) with knowledge of the disease(s) of interest; or (c) of patients with the disease(s) of interest. The Cochrane Handbook for Systematic Reviews [30] recommends the Cooper hierarchy as a useful supplement to more comprehensive checklists such as the NICE checklist.

Two reviewers (HJ and EA for the studies retrieved from the original search and first update search, HJ and DA for the studies retrieved from the second update search) performed quality assessment for included studies, with disagreements resolved by discussion.

### Data extraction

A data extraction form was drafted by one reviewer (HJ) using Microsoft Word (Microsoft Corporation, Washington, US) and checked by a second reviewer (EA). The developed data extraction form was piloted on ten studies. The results of pilot testing were discussed among the team to incorporate any necessary refinements before completion of data collection from all included studies. The formal data extraction was conducted by one reviewer (HJ) and checked by a second reviewer (EA for the original search and first update search, DA for the second update search), with disagreements resolved by discussion. The following information was extracted from all included studies: author, year, country, study objective, method of economic evaluation, intervention and comparator, modelling method, key impacts of antipsychotic medications to include in the model, and rationale for choosing those impacts. The template for data extraction is reported in S2 Table.

### Data analysis

Study characteristics and conclusions were synthesised within a narrative review. No quantitative synthesis was undertaken.

## Results

### Study identification and selection

**Fig 1:**
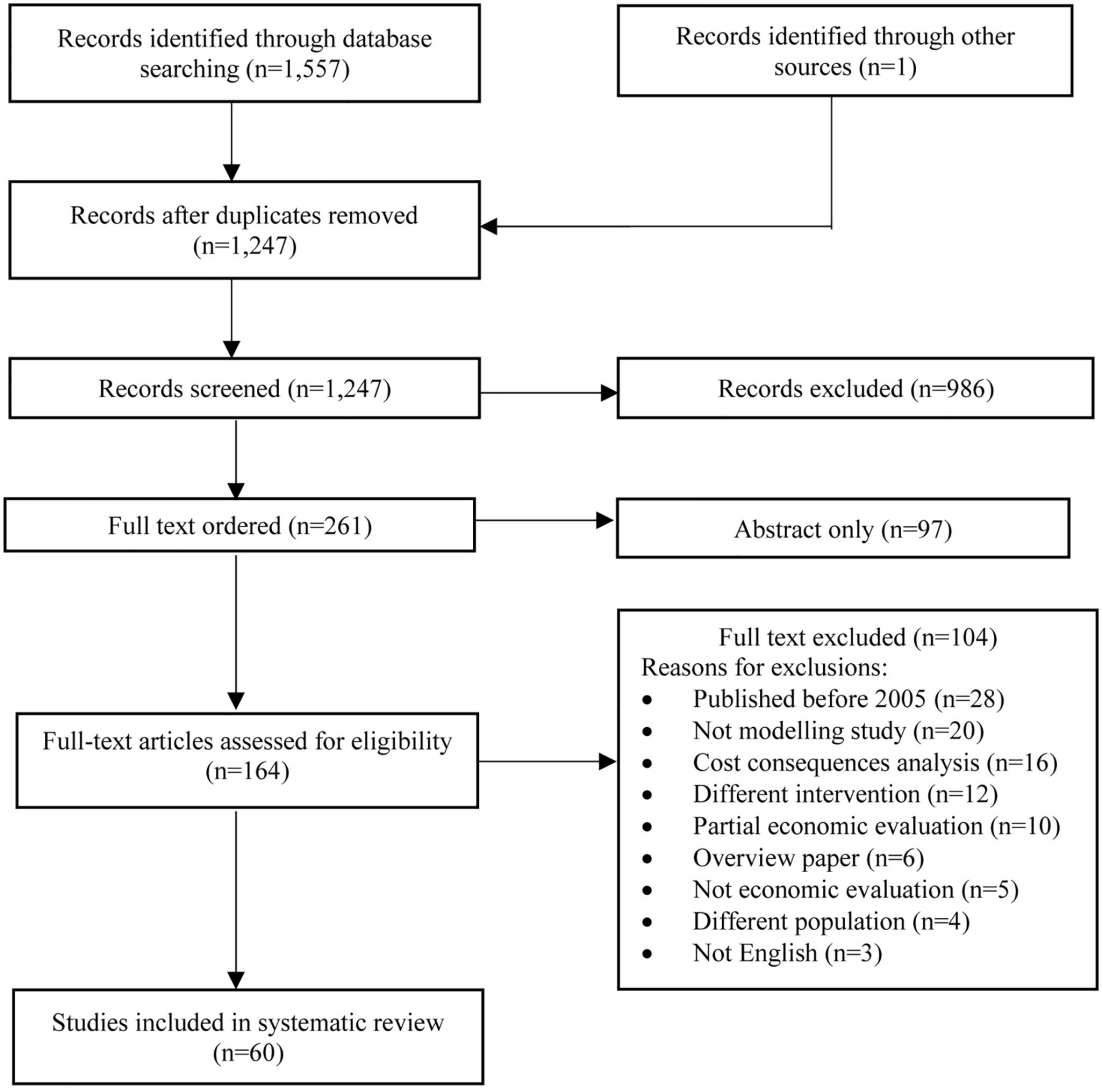
Modified PRISMA flow diagram for the systematic review of health economic models.

A total of 1,557 abstracts were retrieved from electronic searches. The detailed results of the literature search are reported in S1 Table. One eligible model known to the author, but which was not identified by the electronic searches was added to the database. The missing model was reported in one of the chapters of the adult NICE schizophrenia guideline [31] and was not picked up by the electronic search because NICE clinical guidelines have not yet been indexed by mainstream electronic databases. After removing duplicates, 1,247 abstracts remained, 986 of which were excluded for clearly failing to meet at least one inclusion criterion or meeting at least one exclusion criterion, leaving 261 for full-text review. Of these, 97 were abstracts only and for the remaining 164, full articles were retrieved. Of these, sixty papers satisfied the predefined inclusion criteria and were included in the review. A list of excluded studies and reasons for exclusion is reported in S3 Table. The inter-reviewer agreement, measured by Cohen’s kappa was 0.84, which indicates a good level of agreement. A modified PRISMA diagram [32] for the literature selection process is provided in Fig 1.

### Characteristics of included studies

A summary of the characteristics of included studies are reported in Table 1. The characteristics of each individual study is reported in S4 Table.

**Table 1:** Characteristics of included studies assessing antipsychotic medications (n = 60).

	Included studies n (%)
**Population modelled**^1^	
General schizophrenia patients (psychotic status unspecified)	24 (40.0)
Schizophrenia patients in remission	14 (23.3)
Schizophrenia patients in relapse	13 (21.7)
Schizophrenia patients who have history of non-adherence	9 (15.0)
Patients with treatment resistant schizophrenia	2 (3.3)
Schizophrenia patients experiencing adverse events of typicals	2 (3.3)
**Type of economic evaluation**	
CUA	48 (80.0)
CEA excluding CUA	12 (20.0)
**Perspective of cost**	
Healthcare system	31 (51.7)
Third-party payer	19 (31.7)
Society	6 (10.0)
Healthcare system and social care	4 (6.7)
**Clinical effectiveness outcome used by CUA (n = 48)**	
QALYs	43 (89.6)
DALYs	5 (10.4)
**Clinical effectiveness outcome used by CEA excluding CUA (n = 12)**	
Proportion of responders	5 (41.7)
Number of relapses prevented	3 (25.0)
Number of stable months/days	2 (16.7)
Relapse-related hospitalizations	1 (8.3)
PANSS score reductions	1 (8.3)
**Time horizon**	
<1 year	2 (3.3)
1–5 year	45 (75.0)
10-year	4 (6.7)
Lifetime	9 (15.0)
**Modelling techniques adopted**	
Markov model	24 (40.0)
Decision tree	21 (35.0)
Discrete event simulation	9 (15.0)
Microsimulation	5 (8.3)
Not reported	1 (1.7)
**Modelled discontinuation of antipsychotic medication?**	
Yes	55 (91.7)
No	5 (8.3)
**Categories of discontinuation modelled (n = 55)**	
Inefficacy	48 (87.3)
Intolerability	40 (72.7)
Non-adherence	46 (83.6)

**Abbreviations**

CEA: cost-effectiveness analysis; CUA: cost-utility analysis; DALY: disability-adjusted life year; PANSS: the Positive and Negative Syndrome Scale; QALY: quality-adjusted life year.

^1^. Some studies reported results for more than one patient groups. Therefore, the proportion of different population does not add up to 1.

As reported in Table 1, the most commonly modelled populations were general schizophrenia patients (psychotic status unspecified) (24/60, 40.0%), schizophrenia patients in remission (14/60, 23.3%) and in relapse (13/60, 21.7%). Forty-eight of the included studies adopted a CUA approach (80.0%), while twelve studies adopted a CEA approach (20.0%). The key advantage of CUA is that it can capture patients’ health gains (e.g. prevented relapse) and health losses (e.g. adverse events) at the same time, as opposed to CEAs which often capture patients’ health gains only. In terms of time horizon, the majority of identified models adopted a time horizon between one and five years (45/60, 75.0%). Only 15.0% (9/60) adopted a life-time horizon, despite the fact that some of the adverse events of antipsychotics (e.g. weight gain and diabetes) may last for a lifetime. Fifty-five of the sixty studies (91.7%) modelled discontinuation of antipsychotic medication, whilst the remaining five (8.3%) [33–37] assumed that patients would continue to receive their original antipsychotic (i.e. the antipsychotic that patients started with when entering the model) regardless of their treatment response. In clinical practice, discontinuation of antipsychotic medication frequently occurs as a result of inefficacy, intolerability and none-adherence [31] and thus discontinuation is a key event that should be included in economic models. Of the fifty-five studies which modelled discontinuation, the most commonly modelled reasons for discontinuation of antipsychotic were inefficacy (48/55, 87.3%), non-adherence (46/55, 83.6%) and intolerability (40/55, 72.7%).

The antipsychotic modelled by included studies are reported in S5 Table. In total, nineteen different antipsychotic medications (by chemical names) were modelled by all included studies. However, the number of antipsychotic medications included in each study is generally low. As shown in S5 Table, most included studies (43/60, 71.7%) only assessed less than five antipsychotic medications. There is only one study (1.7%) assessed more than 10 antipsychotic medications [38]. The most commonly modelled antipsychotic medications were risperidone (43/60, 71.7%), olanzapine (42/60, 70.0%), paliperidone (22/60, 36.7%) and aripiprazole (17/60, 28.3%). Of the 20 antipsychotics licensed in the UK [2], four antipsychotics (lurasidone, sulpiride, trifluperazine and zuclopenthixol) were only assessed by a small number of studies (≤2), while seven antipsychotics (flupentixol, levomepromazine, periciazine, perphenazine, pimozide, prochlorperazine and promazine) were not assessed by any included studies. In terms of route of administration, 58.3% (35/60) studies assessed oral antipsychotic medications only, 15% (9/60) studies assessed long-acting injectable (LAI) antipsychotic medications only, and 26.7% (16/60) studies assessed both oral and LAI antipsychotic medications.

### Key impacts of antipsychotics modelled by included studies

None of the included studies reported a rationale for selection of the different impacts of antipsychotic medications modelled, including choice of clinical benefits, clinical harms, costs and cost savings. To support understanding of the breadth, perspective and strengths and limitations of economic models, it is valuable to report the impacts of antipsychotic medication that were considered for inclusion in the model, the impacts that were included in the final model and the reason for inclusion or exclusion of each impact. For example, it is useful to know if a key impact was excluded due to lack of data rather than due to irrelevance to the question the model is trying to answer, as the former reason, unlike the latter, highlights a limitation of the model and a gap in the literature.

#### Clinical benefits of antipsychotics

Two types of clinical benefits of using antipsychotics were identified:

1. Reduction of psychotic symptoms (60/60, 100%). All included studies modelled antipsychotics’ effectiveness in reducing psychotic symptoms, but the clinical outcomes that they measured varied and included: risk of relapse (48/60, 80.0%); response rate to antipsychotic (18/60, 30.0%); scores on psychiatric symptom scales (10/60, 16.7%) (e.g. the Brief Psychiatric Rating Scale (BPRS) and the Positive and Negative Syndrome Scale (PANSS)), and number of successfully treated patients (1/60, 1.7%) [39]. Fifteen included studies measured more than one clinical outcome—for example, fourteen studies used response rate to assess an antipsychotic medication’s effectiveness in treating an acute episode of psychosis and risk of relapse to assess an antipsychotic medication’s effectiveness in preventing future relapses. It was noted that even for studies that measured the same outcomes, the definitions of those outcomes or the outcome measures used differed. For example, seven studies defined relapse as exacerbation of psychiatric symptoms which requires hospitalisation, while one study defined relapse as a PANSS score between 20 and 50 [40]. The definitions and/or outcome measures adopted by included studies, are summarised in Table 2.
2. Reduction in mortality (5/60, 8.3%). Mortality was modelled in thirty-one studies (31/60, 51.7%): twenty assumed antipsychotic medications have no impact on patients’ mortality (20/31, 64.5%), five assumed antipsychotic medications reduce patients’ mortality risk (5/31, 16.1%) [41–45], five studies assumed antipsychotic medications increase patients’ mortality risk (5/31, 16.1%) [35, 36, 46–48], and one study assumed antipsychotic medications can both increase and reduce patients’ mortality risk (1/31, 3.2%) [49]. In the six studies which assumed that antipsychotic medications can reduce patients’ mortality risk, they assumed that patients taking antipsychotic medications are less likely to commit suicide and thus have lower mortality.

**Table 2:** Outcomes and measures for assessing psychotic symptoms in included studies.

Outcomes of clinical benefits	Frequency n (%)	Definition/measure of outcome
**1. Risk of relapse**	48 (80.0)	The definitions of relapse adopted by included studies include:
		• Not reported (28/48, 58.3%)
		• Exacerbation of psychiatric symptoms which requires specialized treatment (16/44, 36.4%):
		➢ Hospitalisation or outpatient treatment in specialised facilities (8/16, 50.0%)
		➢ Hospitalisation (7/16, 43.8%)
		➢ Not specified (1/16, 6.3%) [42]
		• Used definition of relapse as adopted by the individual studies included in a meta-analysis (2/48, 4.2%) [38, 50]
		• Patients with PANSS score between 20–50 (1/44, 2.0%) [40]
		• 20% or more decline in PANSS total score and decrease of 1 or more points on the CGI -Severity illness score after week 8 of the trial (1/44, 2.0%) [51]
**2. Response rate to antipsychotic medication**	18 (30.0)	The definitions of response adopted by included studies include:
		• Not reported (10/18, 55.6%)
		• ≥20% reduction from baseline PANSS score (2/18, 11.8%) [52, 53]
		• ≥30% reduction from baseline PANSS score (1/18, 5.9%) Einarson, Pudas [54]
		• ≥30 reduction from baseline PANSS score or CGI—Improvement score of at least two (1/18, 5.9%) [55]
		• ≥40% reduction from baseline PANSS score (1/18, 5.9%) [42]
		• ≥30% improvement from baseline to end point (trial completion) in PANSS, BPRS, or both (1/18, 5.9%) [51]
		• ≥20% reduction from baseline BRPS score or a marked improvement of clinical symptoms such as hallucinations, behavioural problems and suicidal ideation (1/18, 5.9%) [56]
		• Non-relapse or non-rehospitalisation (1/18, 5.9%) [57]
**3. Scores of psychotic symptom scales**	10 (16.7)	The measures for comparing psychotic symptom scores before and after antipsychotic medication include:
		• Standardized mean differences before and after treatment (9/10, 90.0%)
		➢ PANSS (8/9, 88.9%)
		➢ BPRS, PANSS and CGI (1/9, 11.1%)
		• Absolute decrease before and after treatment (1/10, 10.0%): PANSS
**4. Number of successfully treated patients**	1 (1.7)	Successfully treated patients are defined as patients responded to initial treatment and who had no more than two episodes of clinical deterioration and did not need a change of treatment, over the 2 years [39]

**Abbreviations**:

BPRS: Brief Psychiatric Rating Scale; CGI: Clinical Global Impression Scale; PANSS: Positive and Negative Syndrome Scale.

#### Clinical harms of antipsychotics

**Fig 2:**
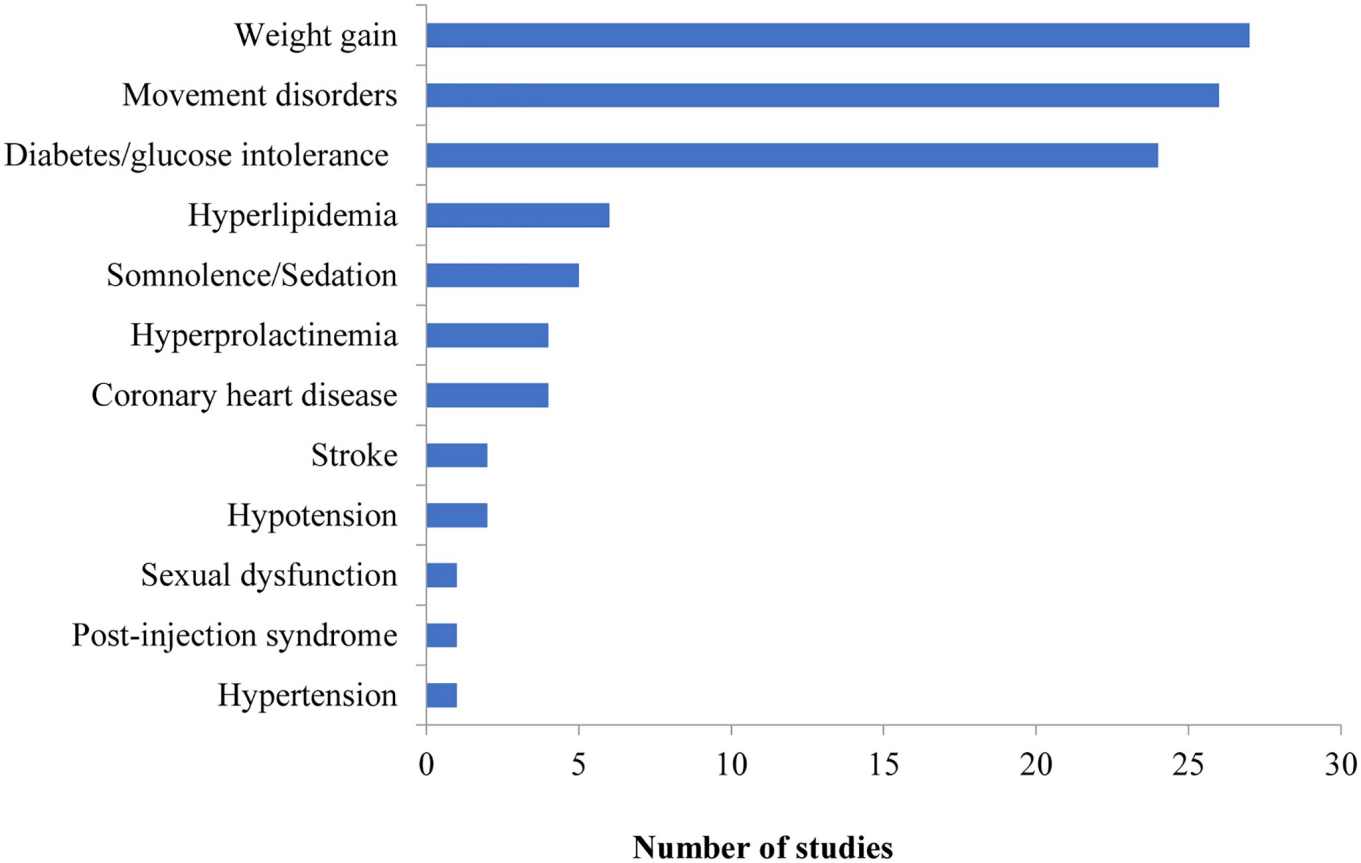
Frequency of adverse events which reduce patients’ health-related quality of life included in identified models.

Two types of clinical harms of antipsychotic medications were identified, both of which are associated with adverse events of antipsychotic medications:

1. Reduction of patient’s utility due to adverse events for CUA studies (27/48, 56.3%). The most frequently modelled adverse events were weight gain (27/27, 100%), movement disorders (26/27, 96.3%), and diabetes/impaired glucose tolerance (24/27, 88.9%). A bar chart showing the frequency of all adverse events modelled is reported in Fig 2.
2. Increase in mortality due to adverse events (6/60, 10.0%). The adverse events which were assumed to increase patients’ mortality risk included diabetes (3/6, 50.0%) [35, 46, 47], cardiovascular disease (3/6, 50.0%) [58–60], weight gain (2/6, 33.3%) [48, 49], agranulocytosis (1/6, 16.7%) [46], hypertension (1/6, 16.7%) [47] and stroke (1/6, 16.7%) [47].

#### Costs associated with antipsychotics

**Fig 3:**
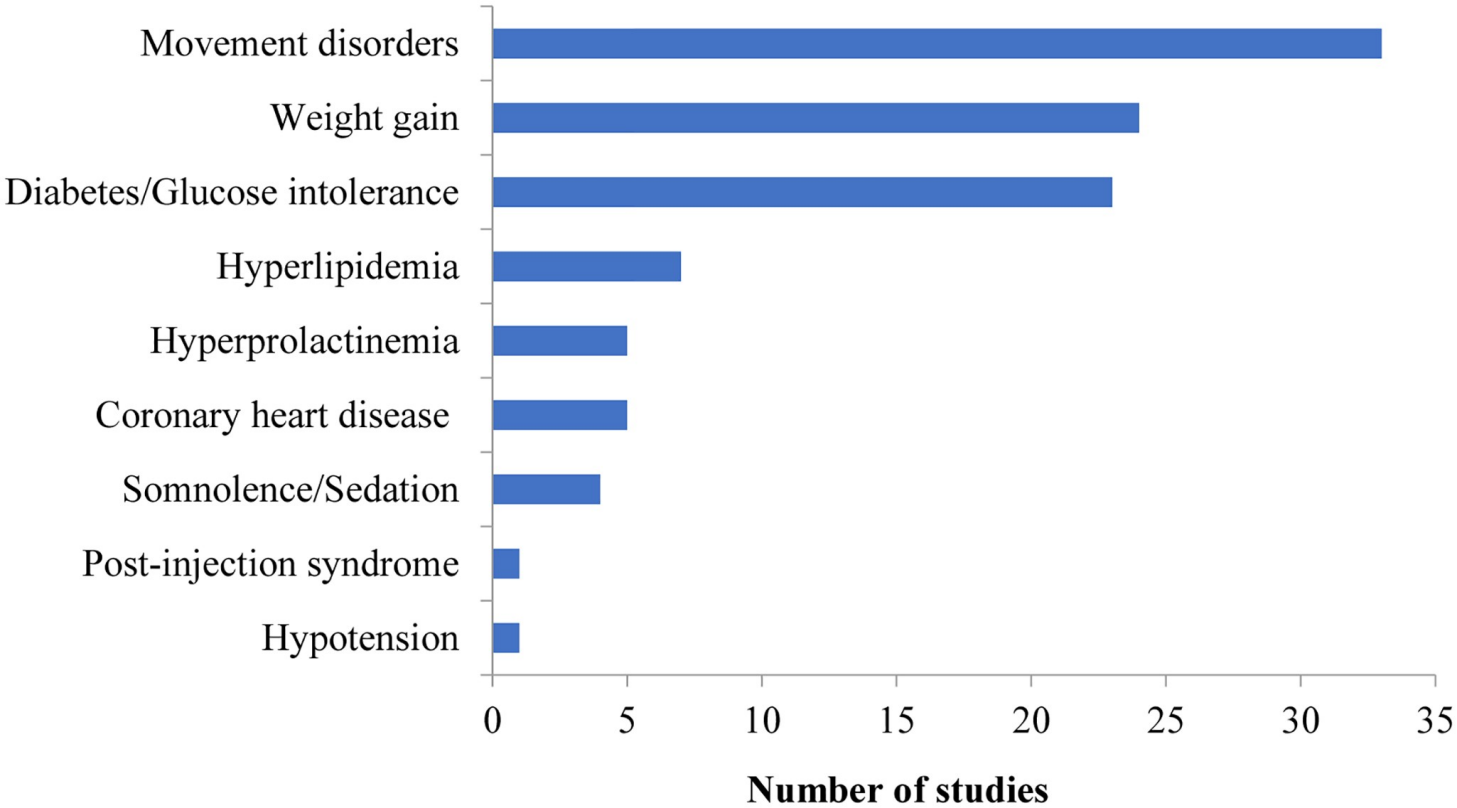
Cost of adverse events considered by identified models.

Two categories of cost related to antipsychotic medication use were modelled by included studies:

1. Acquisition costs of antipsychotic medications (60/60, 100%).
2. Cost of treating adverse events of antipsychotic medications (36/60, 60.0%). Of the thirty-six studies that included the cost of treating adverse events, the most frequently modelled adverse events were movement disorders (33/36, 91.7%), weight gain (24/36, 66.7%) and diabetes/impaired glucose tolerance (23/36, 63.9%). All modelled adverse events and their frequency are shown in Fig 3.

#### Cost savings related to antipsychotics use

The main cost savings resulting from the use of antipsychotic medication related to psychiatric services avoided (58/60, 96.7%). Of the fifty-eight studies that included the cost of psychiatric services, fifty-five studies included inpatient and outpatient/community services (94.8%), one study included the cost of inpatient services only (1.7%) [61], and two studies included the cost of outpatient/community services only (3.4%) [37, 58]. Other cost savings included: sheltered accommodation (12/60, 20.0%), suicide attempts/completion (7/60, 11.7%), productivity losses (6/60, 10.0%), transportation costs (4/60, 6.7%) [35, 62–64] and meal costs (1/60, 1.7%) [64].

### Quality assessment

The performance of included studies on all items of Section 2 of the NICE checklist and the Cooper hierarchy are reported in S6 and S7 Tables, respectively, and are briefly summarised below.

#### NICE checklist

According to the quality assessment results of the NICE checklist, fifty-four studies were deemed to have very serious limitations (90.0%), four were deemed to have potentially serious limitations (6.7%) [41, 64–66], and two were deemed to have minor limitations (3.3%) [38, 50]. Key problems identified included: (1) resource use data not obtained from the best available source (44/60, 73.3%), such as prospective data collection or analysis of reliable administrative data for specifically conducted for the study; (2) failure to include all important and relevant costs (38/60, 63.3%), e.g. cost of treating adverse events of antipsychotic medications; (3) failure to include all important and relevant clinical outcomes (35/60, 58.3%), e.g. disutility caused by adverse events of antipsychotics; and (4) treatment effects data not obtained from the best available source (28/60, 46.7%), such as meta-analysis of RCTs or single high-quality RCT.

#### Cooper hierarchy

Of the six categories included in the Cooper hierarchy [23], three of them (adverse events, resources use and costs) may include multiple data inputs (i.e. more than one data source can be used for that category). For these three categories, the score of the lowest quality evidence is reported. Most studies used high ranked evidence for clinical treatment effects (42/60, 70.0%) and unit costs (43/60, 71.7%), medium-ranked evidence for baseline clinical events (43/60, 71.7%), and low-ranked evidence for resource use (50/60, 83.3%) and adverse events (29/53, 48.3%). Of the 48 CUA studies which modelled patients’ utilities, most used medium ranked evidence to inform utility estimates (46/48, 95.8%).

### Good practices for modelling the cost-effectiveness of antipsychotic medications

Based on findings of this review and the hierarchy of evidence suggested by Cooper *et al*. [23], good practices for modelling the cost-effectiveness of antipsychotic medications are derived and summarised in Table 3. The suggested good practices cover general modelling methods (e.g. choice of intervention and time horizon, and key event to model), key impacts of antipsychotic medication to include in the model (e.g. clinical benefits and harms, and costs and cost savings), and source of input data. It is recommended that the suggested good practices should be used by modellers in conjunction with their own judgement, taking into consideration of specific decision-making context and practical resource constrains.

**Table 3:** Good practices for modelling the cost-effectiveness of antipsychotic medications.

Element	Recommendations
***General modelling methods***	
**Intervention & Comparator**	Include all relevant antipsychotic medications.
**Type of economic evaluation**	Cost-utility analysis (CUA) is recommended, as it can capture patients’ health gains (e.g. prevented relapse) and health loss (e.g. adverse events) at the same time.
**Time horizon**	A life-time horizon is recommended, as some of the adverse events of antipsychotic medications (e.g. weight gain and diabetes) may last for a lifetime.
**Key event to model**	Discontinuation of antipsychotic medication due to different reasons need to be modelled. A non-exhaustive list of reasons for discontinuation includes inefficacy, intolerability and non-adherence.
***Key impacts of antipsychotic medication to include in the model***	
**Justify choice of key impacts of antipsychotic medications to include in the model**	Report the rationale for choosing impacts of antipsychotic medication to model, including:
	(1) what impacts of antipsychotic medication were considered for inclusion in the model;
	(2) which impacts were included in the final model;
	(3) reason for inclusion/exclusion for each impact.
**Clinical benefits**	(1) Reduction in psychotic symptoms;
	(2) Reduction in patients’ risk of mortality due to suicide.
**Clinical harms**	(1) Disutility due to adverse events*;
	(2) Increased mortality due to adverse events*.
**Costs**	(1) Cost of providing antipsychotic medication (including drug cost, and administration cost and monitoring cost if applicable);
	(2) Cost of treating adverse events*.
**Cost savings**	Inpatient and outpatient psychiatric services. If a societal perspective is adopted, other relevant cost savings need to be included. A non-exhaustive list includes sheltered accommodation, suicide attempts/completion, productivity losses, transportation costs and meal costs.
***Source of input data*** [23]	
**Baseline clinical data**	Analysis of reliable local databases.
**Clinical effect size and adverse events data**	Network meta-analysis of randomised clinical trials covering all (or as many as possible) antipsychotic mediations of interest, measuring final outcomes.
**Unit cost data**	Unit cost calculated based on reliable local database.
**Resource use data**	Analysis of reliable local databases.
**Utility data**	Direct utility assessment from a sample either of the general population but with knowledge of the disease(s) of interest; or patients with schizophrenia.

* Choice of adverse events to include should be based on i) the adverse event profile of the antipsychotic medication(s) in question and ii) the expected magnitude of the health and cost impact of the adverse events.

## Discussion

### Summary of findings

This review found that the key impacts of antipsychotic medications considered by existing models are:

- *clinical benefits*: reduction in psychotic symptoms and reduction in patients’ risk of suicide;
- *clinical harms*: the impact of adverse events on patients’ health-related quality of life and mortality;
- *costs*: cost of antipsychotic medication, and cost of treating adverse events of antipsychotic medication;
- *cost savings*: inpatient and outpatient psychiatric services.

The results of this review suggest that models assessing antipsychotic medications were subject to considerable inconsistencies, particularly with respect to two aspects of the analyses: (1) clinical outcomes used for assessing reduction in psychotic symptoms, which included response rate, risk of relapse, scores on psychiatric symptom scales and number of successfully treated patients; and (2) types of adverse events considered in the model. One possible explanation for the variation in choice of adverse events is that different antipsychotic medications are associated with different adverse event profiles. For example, compared to first-generation antipsychotic medications (e.g. haloperidol), second-generation antipsychotic medications (e.g. olanzapine) are less likely to cause EPS but more likely to cause weight gain and diabetes. Therefore, models which only assess second-generation antipsychotic medications may choose to include weight gain and diabetes, but not EPS. However, even for studies assessing the same antipsychotic medications, the choice of adverse events still differed.

In addition, the review found the quality of existing models assessing antipsychotic medication is generally low. Common reasons for low-quality included failure to capture the health and cost impact of adverse events of antipsychotic medications, input data not obtained from the best available source and potential conflicts of interest. In order to improve the consistency and quality of future models in antipsychotic medications, good practices are suggested based on the findings of this review.

### Strengths & limitations

#### Strengths

This study has three main strengths. Firstly, to our knowledge, this is the first systematic review which explores the key impacts of antipsychotic medications considered by existing health economic models. Secondly, based on the findings of this review, good practice for modelling antipsychotic medications are suggested. These good practice recommendations can be used to improve the consistency and methodological and reporting quality of future health economic models assessing antipsychotic medications. Thirdly, a number of strategies were devised to help ensure that relevant studies were not missed, including checking key papers and the publications of key health economists, and checking references of published systematic reviews.

#### Limitations

This review is subject to three main limitations. First, only models using a CEA or CUA approach were included. Models using other approaches, such as cost-benefit analysis (CBA), cost-consequences analysis (CCA) and cost-minimisation analysis (CMA), were excluded. However, it has been reported that CBA, CCA and CMA have problems with their reliability, validity and even morality [67, 68], and are therefore less frequently used in the health care sector compared to CEA and CUA. Second, this review only included models published after 2005. This is because studies published before that time were deemed to have limited relevance to current practice due to the rapidly changing nature of treatments, health services and methods of economic evaluation. Third, development of good practice based on previous models may be subject to the limitations of those models. For example, key impacts of antipsychotic medication may have been missed if all existing models failed to include them. However, these studies should at least provide a starting point for understanding key disease-related factors and the impact(s) of treatments upon that disease [69].

## Supporting information

S1 Text Electronic search strategies. (DOCX)

S1 Table Systematic review protocol. (DOCX)

S2 Table Template for data extraction. (DOCX)

S3 Table List of excluded studies with reasons. (DOCX)

S4 Table Characteristics of included studies. (XLSX)

S5 Table Number and type of antipsychotic medications covered by included studies. (DOCX)

S6 Table Performance of included studies assessed by Section 2 of the NICE checklist. (DOCX)

S7 Table Performance of included studies assessed by the Cooper hierarchy. (DOCX)

S1 Checklist PRISMA 2009 checklist. (DOC)
