# Supplementary material for: Systematic review of the methods of health economic models assessing antipsychotic medication for schizophrenia
Source: PLoS One. 2020 Jul 10;15(7):e0234996. doi: 10.1371/journal.pone.0234996 (PMC7351140; doi:10.1371/journal.pone.0234996)
Supplement: S1 Text — (DOCX) [file pone.0234996.s001.docx]

**S1 Text. Electronic search strategies**

**1. MEDLINE search strategy**

Database used for the original search:

- Ovid MEDLINE(R) 1946 to March Week 4 2018,
- Ovid MEDLINE(R) In-Process & Other Non-Indexed Citations April 02, 2018

|  | **Search terms** | **Results** |
| --- | --- | --- |
| 1 | exp "schizophrenia spectrum and other psychotic disorders"/ | 136760 |
| 2 | exp psychotic disorders/ or exp perceptual disorders/ or delusions/ or hallucinations/ or speech disorders/ or catatonia/ or paranoid disorders/ | 90516 |
| 3 | (at risk mental state or clinical high risk or ultra high risk or psychos?s risk syndrome$ or attenuated psychos?s syndrome).mp. | 1533 |
| 4 | ((at risk or high risk or prodrom$ or earl$ or subclinic$ or preclinic$ or subthreshold or onset or transition$ or convert$) adj2 (psychos?s or psychotic or schizo$)).mp. | 7117 |
| 5 | (schizo$ or psychotic$ or psychosis or psychoses or ((thinking or thought) adj2 (disorder$ or disturbance$ or problem$)) or delusion$ or catatoni$ or hallucinat$ or hebephreni$ or oligophreni$ or paranoi$).mp. | 224440 |
| 6 | ((chronic$ or long term or persistent or serious$ or sever$) adj2 (mental$ or psychiatric or psycho$) adj2 (ill$ or disorder$ or disease$ or problem$ or disturb$ or disable$)).mp. | 15378 |
| 7 | 1 or 2 or 3 or 4 or 5 or 6 | 259790 |
| 8 | exp Cost-Benefit Analysis/ | 72027 |
| 9 | (cost$ adj2 (effect$ or benefit$ or utility or utilities or outcome$ or consequence$)).mp. | 166166 |
| 10 | (cost$ adj minimi$).mp. | 1229 |
| 11 | 8 or 9 or 10 | 166732 |
| 12 | exp Decision Theory/ or exp Decision Making, Computer-Assisted/ or Decision Support Systems, Management/ or exp Decision Making/ or Decision Support Systems, Clinical/ or Decision Trees/ or Decision Making, Organizational/ or exp Decision Support Techniques/ | 388496 |
| 13 | Computer Simulation/ or Patient Simulation/ or models, theoretical/ or exp models, organizational/ or exp models, statistical/ or exp models, economic/ or monte carlo method/ or Markov Chains/ | 657020 |
| 14 | (decision adj (tree$ or analysis or analyses or analytic$ or support)).mp. | 46519 |
| 15 | ((disease or mathematical or optimization or optimisation or decision$ or economic$ or pharmacoeconomic or simulation or cohort or Markov or Markov chain or state transition or patient level or individual level or individual sampling or event history or agent based) adj model$).mp. | 362442 |
| 16 | ((discrete event or discrete individual or agent based or hybrid or inverse or monte carlo or real time) adj simulation).mp. | 9837 |
| 17 | (system dynamics or DES).mp. | 349782 |
| 18 | 12 or 13 or 14 or 15 or 16 or 17 | 1676906 |
| 19 | 7 and 11 and 18 | 290 |
| 20 | (letter or news or editorial or historical article).pt. | 1935389 |
| 21 | 19 not 20 | 286 |
| 22 | exp animals/ not humans/ | 4435919 |
| 23 | 21 not 22 | 276 |
| 24 | limit 23 to english language | 247 |

**2. EMBASE search strategy**

Database used for the original search: EMBASE Classic & EMBASE (1947 to 2018 week 14)

|  | **Search terms** | **Results** |
| --- | --- | --- |
| 1 | exp schizophrenia/ | 179615 |
| 2 | exp psychosis/ or exp thinking disorder/ or exp delusion/ or exp hallucination/ or exp speech disorder/ or catatonia/ or hebephrenia/ or oligophrenia/ or paranoia/ | 749574 |
| 3 | (at risk mental state or clinical high risk or ultra high risk or psychos?s risk syndrome$ or attenuated psychos?s syndrome).mp. | 3261 |
| 4 | ((at risk or high risk or prodrom$ or earl$ or subclinic$ or preclinic$ or subthreshold or onset or transition$ or convert$) adj2 (psychos?s or psychotic or schizo$)).mp. | 12366 |
| 5 | (schizo$ or psychotic$ or psychosis or psychoses or ((thinking or thought) adj2 (disorder$ or disturbance$ or problem$)) or delusion$ or catatoni$ or hallucinat$ or hebephreni$ or oligophreni$ or paranoi$).mp. | 350393 |
| 6 | ((chronic$ or long term or persistent or serious$ or sever$) adj2 (mental$ or psychiatric or psycho$) adj2 (ill$ or disorder$ or disease$ or problem$ or disturb$ or disable$)).mp. | 21514 |
| 7 | 1 or 2 or 3 or 4 or 5 or 6 | 824647 |
| 8 | *economic evaluation/ or exp "cost benefit analysis"/ or exp "cost effectiveness analysis"/ or exp "cost minimization analysis"/ or exp "cost utility analysis"/ | 206729 |
| 9 | (cost$ adj2 (effect$ or benefit$ or utility or utilities or outcome$ or consequence$)).mp. | 293914 |
| 10 | (cost$ adj minimi$).mp. | 3978 |
| 11 | 8 or 9 or 10 | 297220 |
| 12 | exp decision support system/ or decision making/ or "decision tree"/ or clinical decision making/ or decision theory/ or medical decision making/ | 334710 |
| 13 | exp simulation/ or computer model/ or individual based population model/ or population model/ or exp mathematical model/ or stochastic model/ or exp disease model/ or hidden Markov model/ or statistical model/ | 669682 |
| 14 | (decision adj (tree$ or analysis or analyses or analytic$ or support)).mp. [mp=title, abstract, heading word, drug trade name, original title, device manufacturer, drug manufacturer, device trade name, keyword] | 47638 |
| 15 | ((disease or mathematical or optimization or optimisation or decision$ or economic$ or pharmacoeconomic or simulation or cohort or Markov or Markov chain or state transition or patient level or individual level or individual sampling or event history or agent based) adj model$).mp. [mp=title, abstract, heading word, drug trade name, original title, device manufacturer, drug manufacturer, device trade name, keyword] | 277684 |
| 16 | ((discrete event or discrete individual or agent based or hybrid or inverse or monte carlo or real time) adj simulation).mp. [mp=title, abstract, heading word, drug trade name, original title, device manufacturer, drug manufacturer, device trade name, keyword] | 12330 |
| 17 | (system dynamics or DES).mp. [mp=title, abstract, heading word, drug trade name, original title, device manufacturer, drug manufacturer, device trade name, keyword] | 491078 |
| 18 | 12 or 13 or 14 or 15 or 16 or 17 | 1538100 |
| 19 | 7 and 11 and 18 | 1154 |
| 20 | (letter or editorial or note).pt. | 2279668 |
| 21 | 19 not 20 | 1053 |
| 22 | animal/ | 1838994 |
| 23 | exp animal experiment/ | 2204175 |
| 24 | nonhuman/ | 5393773 |
| 25 | (rat or rats or mouse or mice or hamster or hamsters or animal or animals or dog or dogs or cat or cats or bovine or sheep).ti,ab,sh. | 6050564 |
| 26 | 22 or 23 or 24 or 25 | 8800912 |
| 27 | exp human/ | 19609898 |
| 28 | human experiment/ | 402383 |
| 29 | 27 or 28 | 19611482 |
| 30 | 26 not (26 and 29) | 6648319 |
| 31 | 21 not 30 | 1020 |
| 32 | limit 31 to english language | 964 |

**3. PsycINFO search strategy**

Database used for the original search: PsycINFO (1806 to March Week 4 2018)

|  | **Search terms** | **Results** |
| --- | --- | --- |
| 1 | exp Schizophrenia/ | 84386 |
| 2 | exp psychosis/ or exp thought disturbances/ or exp delusions/ or exp hallucinations/ or exp speech disorders/ or exp catatonia/ or exp paranoia/ | 138246 |
| 3 | (at risk mental state or clinical high risk or ultra high risk or psychos?s risk syndrome$ or attenuated psychos?s syndrome).mp. | 1579 |
| 4 | ((at risk or high risk or prodrom$ or earl$ or subclinic$ or preclinic$ or subthreshold or onset or transition$ or convert$) adj2 (psychos?s or psychotic or schizo$)).mp. | 8313 |
| 5 | (schizo$ or psychotic$ or psychosis or psychoses or ((thinking or thought) adj2 (disorder$ or disturbance$ or problem$)) or delusion$ or catatoni$ or hallucinat$ or hebephreni$ or oligophreni$ or paranoi$).mp. | 195990 |
| 6 | ((chronic$ or long term or persistent or serious$ or sever$) adj2 (mental$ or psychiatric or psycho$) adj2 (ill$ or disorder$ or disease$ or problem$ or disturb$ or disable$)).mp. | 20652 |
| 7 | 1 or 2 or 3 or 4 or 5 or 6 | 232113 |
| 8 | (cost$ adj2 (effect$ or benefit$ or utility or utilities or outcome$ or consequence$)).mp. | 22966 |
| 9 | (cost$ adj minimi$).mp. | 137 |
| 10 | 8 or 9 | 23069 |
| 11 | exp Decision Support Systems/ or exp Decision Making/ or exp Decision Theory/ or exp Management Decision Making/ | 97733 |
| 12 | exp simulation/ or models/ | 111446 |
| 13 | (decision adj (tree$ or analysis or analyses or analytic$ or support)).mp. | 7011 |
| 14 | ((disease or mathematical or optimization or optimisation or decision$ or economic$ or pharmacoeconomic or simulation or cohort or Markov or Markov chain or state transition or patient level or individual level or individual sampling or event history or agent based) adj model$).mp. | 22754 |
| 15 | ((discrete event or discrete individual or agent based or hybrid or inverse or monte carlo or real time) adj simulation).mp. | 1493 |
| 16 | (system dynamics or DES).mp. | 31679 |
| 17 | 11 or 12 or 13 or 14 or 15 or 16 | 242172 |
| 18 | 7 and 10 and 17 | 144 |
| 19 | (editorial or letter or dissertation or abstract).dt. | 521331 |
| 20 | 18 not 19 | 140 |
| 21 | (animal or animals or rat or rats or mouse or mice or hamster or hamsters or dog or dogs or cat or cats or bovine or sheep or ovine or pig or pigs).ab,ti,id,de. | 331981 |
| 22 | 20 not 21 | 132 |
| 23 | limit 22 to english language | 121 |

**4. NHS Economic Evaluation Database (NHSEED) and the Health Technology Assessment Database (HTA) search strategy**

Database used for the original search: NHSEED and HTA accessed via Cochrane library interface (<http://onlinelibrary.wiley.com/cochranelibrary/search>) on 23/June/2015

|  | **Search terms** | **Results** |
| --- | --- | --- |
| 1 | MeSH DESCRIPTOR Schizophrenia Spectrum and Other Psychotic Disorders EXPLODE ALL TREES IN NHSEED,HTA | 286 |
| 2 | MeSH DESCRIPTOR Perceptual Disorders EXPLODE ALL TREES IN NHSEED,HTA | 7 |
| 3 | MeSH DESCRIPTOR Delusions EXPLODE ALL TREES IN NHSEED,HTA | 1 |
| 4 | MeSH DESCRIPTOR Hallucinations EXPLODE ALL TREES IN NHSEED,HTA | 5 |
| 5 | MeSH DESCRIPTOR Speech Disorders EXPLODE ALL TREES IN NHSEED,HTA | 23 |
| 6 | MeSH DESCRIPTOR Catatonia EXPLODE ALL TREES IN NHSEED,HTA | 3 |
| 7 | MeSH DESCRIPTOR Paranoid Disorders EXPLODE ALL TREES IN NHSEED,HTA | 0 |
| 8 | (at risk mental state or clinical high risk or ultra high risk or psychos*s risk syndrome* or attenuated psychos*s syndrome) IN NHSEED, HTA | 1 |
| 9 | ((at risk or high risk or prodrom* or earl* or subclinic* or preclinic* or subthreshold or onset or transition* or convert*) adj2 (psychos*s or psychotic or schizo*)) IN NHSEED, HTA | 14 |
| 10 | ((schizo* or psychotic* or psychosis or psychoses or ((thinking or thought) adj2 (disorder* or disturbance* or problem*)) or delusion* or catatoni* or hallucinat* or hebephreni* or oligophreni* or paranoi*)) IN NHSEED, HTA | 432 |
| 11 | (((chronic* or long term or persistent or serious* or sever*) adj2 (mental* or psychiatric or psycho*) adj2 (ill* or disorder* or disease* or problem* or disturb* or disable*))) IN NHSEED, HTA | 95 |
| 12 | #1 OR #2 OR #3 OR #4 OR #5 OR #6 OR #7 OR #8 OR #9 OR #10 OR #11 | 510 |
| 13 | MeSH DESCRIPTOR Decision Support Techniques EXPLODE ALL TREES IN NHSEED,HTA | 1314 |
| 14 | MeSH DESCRIPTOR Decision Making EXPLODE ALL TREES IN NHSEED,HTA | 266 |
| 15 | MeSH DESCRIPTOR Decision Making, Computer-Assisted EXPLODE ALL TREES IN NHSEED,HTA | 338 |
| 16 | MeSH DESCRIPTOR Decision Making, Organizational EXPLODE ALL TREES IN NHSEED,HTA | 13 |
| 17 | MeSH DESCRIPTOR Decision Support Systems, Clinical EXPLODE ALL TREES IN NHSEED,HTA | 50 |
| 18 | MeSH DESCRIPTOR Decision Support Systems, Management EXPLODE ALL TREES IN NHSEED,HTA | 1 |
| 19 | MeSH DESCRIPTOR Decision Theory EXPLODE ALL TREES IN NHSEED,HTA | 857 |
| 20 | MeSH DESCRIPTOR Computer Simulation IN NHSEED,HTA | 468 |
| 21 | MeSH DESCRIPTOR Patient Simulation IN NHSEED,HTA | 13 |
| 22 | MeSH DESCRIPTOR Models, Economic EXPLODE ALL TREES IN NHSEED,HTA | 1990 |
| 23 | MeSH DESCRIPTOR Models, Theoretical EXPLODE ALL TREES IN NHSEED,HTA | 3159 |
| 24 | MeSH DESCRIPTOR Markov Chains EXPLODE ALL TREES IN NHSEED,HTA | 2018 |
| 25 | MeSH DESCRIPTOR Monte Carlo Method EXPLODE ALL TREES IN NHSEED,HTA | 414 |
| 26 | ((decision adj (tree* or analysis or analyses or analytic* or support))) IN NHSEED, HTA | 3608 |
| 27 | (((disease or mathematical or optimization or optimisation or decision* or economic* or pharmacoeconomic or simulation or cohort or Markov or Markov chain or state transition or patient level or individual level or individual sampling or event history or agent based) adj model*)) IN NHSEED, HTA | 5705 |
| 28 | (((discrete event or discrete individual or agent based or hybrid or inverse or monte carlo or real time) adj simulation)) IN NHSEED, HTA | 856 |
| 29 | ((system dynamics or DES)) IN NHSEED, HTA | 625 |
| 30 | #13 OR #14 OR #15 OR #16 OR #17 OR #18 OR #19 OR #20 OR #21 OR #22 OR #23 OR #24 OR #25 OR #26 OR #27 OR #28 OR #29 | 8891 |
| 31 | #12 AND #30 | 130 |
| 32 | (English:lp) IN NHSEED, HTA | 21864 |
| 33 | #31 AND #32 | 91 |
