# Supplementary material for: Systematic review of the methods of health economic models assessing antipsychotic medication for schizophrenia
Source: PLoS One. 2020 Jul 10;15(7):e0234996. doi: 10.1371/journal.pone.0234996 (PMC7351140; doi:10.1371/journal.pone.0234996)
Supplement: S1 Table — (DOCX) [file pone.0234996.s002.docx]

**S1 Table. Systematic review protocol**

| **Review question** | What health and cost impacts of antipsychotic medication have been considered by existing health economic models? |
| --- | --- |
| **Objectives** | 1. Identify which clinical benefits, clinical harms, costs and cost savings of antipsychotic medication have been considered by existing models. 2. Assess quality of existing models. 3. Suggest good practice recommendations for future economic models of antipsychotic medications. |
| **Inclusion criteria** | Population: Young people (under 18 years of age) and/or adults (18 years and older) with a non-specific diagnosis of psychosis or with a diagnosis of schizophrenia (including schizoaffective disorder and delusional disorder).  Subgroups: None.  Interventions: Antipsychotic medication(s).  Comparators: Antipsychotic medication(s) or placebo or nothing.  Outcomes: No limits.  Study design: Model-based economic evaluation adopting either a cost-effectiveness analysis (CEA) or cost-utility analysis (CUA) approach. |
| **Exclusion criteria** | - Reviews, commentaries, letters, editorials, or abstracts (published systematic reviews will be excluded, but their reference lists will be checked) - Published before 2005. - Not reported in English |
| **Database** | - MEDLINE (including in-Process & other non-indexed), access through the Ovid interface (https://ovidsp.ovid.com/). - EMBASE, access through the Ovid interface (https://ovidsp.ovid.com/). - PsycINFO, access through the Ovid interface (https://ovidsp.ovid.com/). - The NHS Economic Evaluation Database (NHS EED), access through the Cochrane library interface. - The Health Technology Assessment (HTA) Database, access through the Cochrane library interface. |
| **Review strategy** | The methodological quality of each study will be assessed using Section 2 of the NICE checklist [1] and the Cooper hierarchy [2] |

**References**

- 1. National Institute for Health and Care Excellence. Developing NICE guidelines: the manual. London, UK: National Institute for Health and Care Excellence; 2014.
- 2. Cooper N, Coyle D, Abrams K, Mugford M, Sutton A. Use of evidence in decision models: An appraisal of health technology assessments in the UK since 1997. Journal of Health Services Research and Policy. 2005;10(4):245-50.
