# Supplementary material for: Systematic review of the methods of health economic models assessing antipsychotic medication for schizophrenia
Source: PLoS One. 2020 Jul 10;15(7):e0234996. doi: 10.1371/journal.pone.0234996 (PMC7351140; doi:10.1371/journal.pone.0234996)
Supplement: S2 Table — (DOCX) [file pone.0234996.s003.docx]

**S2 Table. Template for data extraction**

| ***Studies assessing antipsychotic medications*** |  |  |
| --- | --- | --- |
| **General information** |  |  |
| First author name | String | N/A |
| Publication year | String | N/A |
| Geographical setting | Category | By country |
| **Design of economic evaluation** |  |  |
| Method of economic evaluation | Category | CUA, CEA |
| Perspective of economic evaluation | Category | Healthcare system, healthcare system and social care, third-party payer, and societal perspective |
| Population modelled | String | N/A |
| Modelling techniques used | Category | By modelling technique |
| **Type of intervention covered** |  |  |
| Route of administration | Category | Standard oral, oral extended release, oral disintegrating tablet, and long-acting injectable |
| Type of antipsychotic medication | Category | By chemical name |
| **Design of model** |  |  |
| Time horizon | String | N/A |
| Final effectiveness outcome | String | N/A |
| Discontinuation of antipsychotic medication modelled? | Category | Yes/No |
| Reason for discontinuation of antipsychotic medication | Category | Inefficacy, intolerability, non-adherence and other reasons |
| **Report the rationale for choosing impacts of antipsychotic medication to model** | | |
| Report the rationale for choosing impacts of antipsychotic medication to model? | Category | Yes/No |
| **Impacts of antipsychotic medication** |  |  |
| Type of clinical benefits modelled | String | N/A |
| Type of clinical harms modelled | String | N/A |
| Cost of antipsychotic medication | String | N/A |
| Cost savings due to use of antipsychotic medication | String | N/A |
