# Supplementary material for: Systematic review of the methods of health economic models assessing antipsychotic medication for schizophrenia
Source: PLoS One. 2020 Jul 10;15(7):e0234996. doi: 10.1371/journal.pone.0234996 (PMC7351140; doi:10.1371/journal.pone.0234996)
Supplement: S3 Table — (DOCX) [file pone.0234996.s004.docx]

**S3 Table. List of excluded studies with reasons for exclusion**

| **Number** | **Study** | **Primary reason for exclusion^1^** |
| --- | --- | --- |
| 1 | Alexeyeva *et al*, 2001 [1] | Published before 2005 |
| 2 | Almond *et al*, 1998 [2] | Published before 2005 |
| 3 | Almond *et al*, 2000 [3] | Published before 2005 |
| 4 | Andrews *et al*, 2003 [4] | Partial economic evaluation |
| 5 | Annemans *et al*, 2012 [5] | Partial economic evaluation |
| 6 | Arteaga Duarte *et al*, 2019 [6] | Not English |
| 7 | Barnett *et al*, 2012 [7] | Not modelling study |
| 8 | Barnett *et al*, 2015 [8] | Different population |
| 9 | Basu *et al*, 2018 [9] | Not economic evaluation |
| 10 | Bera *et al*., 2014 [10] | Cost consequences analysis |
| 11 | Bettinger *et al*, 2007 [11] | Not modelling study |
| 12 | Bobes *et al*, 2004 [12] | Published before 2005 |
| 13 | Bouvy *et al*, 2012 [13] | Different population |
| 14 | Byrom *et al*, 1998 [14] | Published before 2005 |
| 15 | Carswell *et al*, 2010 [15] | Not modelling study |
| 16 | Colombo *et al*, 2008 [16] | Cost consequences analysis |
| 17 | Davies *et al*, 1993 [17] | Published before 2005 |
| 18 | Davies *et al*, 1998 [18] | Published before 2005 |
| 19 | Davies *et al*, 2007 [19] | Not modelling study |
| 20 | Davies *et al*, 2008 [20] | Not modelling study |
| 21 | de Menil *et al*, 2015 [21] | Not modelling study |
| 22 | Dickey *et al*, 2004 [22] | Not modelling study |
| 23 | Druais *et al*, 2017 [23] | Not English |
| 24 | Duggan *et al*, 2003 [24] | Published before 2005 |
| 25 | Edwards *et al*, 2005 a [25] | Cost consequences analysis |
| 26 | Edwards *et al*, 2005 b [26] | Cost consequences analysis |
| 27 | Edwards *et al*, 2008 [27] | Cost consequences analysis |
| 28 | Edwards *et al*, 2012 [28] | Cost consequences analysis |
| 29 | Einarson *et al*, 2013 [29] | Not modelling study |
| 30 | Emsley *et al*, 2004 [30] | Published before 2005 |
| 31 | Frey *et al*, 2014 [31] | Cost consequences analysis |
| 32 | Ganguly *et al*, 2003 [32] | Published before 2005 |
| 33 | Girardin *et al,* 2014 [33] | Different intervention |
| 34 | Girardin *et al,* 2019 [34] | Different intervention |
| 35 | Glazer *et al*, 1996 [35] | Published before 2005 |
| 36 | Glennie *et al*, 1997 [36] | Published before 2005 |
| 37 | Gozlan *et al*, 2018 [37] | Not English |
| 38 | Grande *et al*, 2020 [38] | Review paper |
| 39 | Greenhalgh *et al*, 2005 [39] | Different intervention |
| 40 | Grieve *et al*, 2008 [40] | Not modelling study |
| 41 | Gutierrez-Recacha *et al*, 2006 [41] | Different intervention |
| 42 | Haby *et al*, 2004 [42] | Published before 2007 |
| 43 | Hansen *et al*, 2002 [43] | Published before 2005 |
| 44 | Heeg *et al*, 2005 [44] | Cost consequences analysis |
| 45 | Henrique *et al*, 2020 [45] | Review paper |
| 46 | Janssen *et al*, 2011 [46] | Not modelling study |
| 47 | Jin *et al,* 2019 [47] | Different intervention |
| 48 | Johnson-Masotti *et al*, 2000 [48] | Different population |
| 49 | Joshi *et al*, 2015 [49] | Partial economic evaluation |
| 50 | Kaaya *et al*, 2013 [50] | Review paper |
| 51 | Karki *et al*, 2001 [51] | Not modelling study |
| 52 | Keks *et al*, 1997 [52] | Review paper |
| 53 | Kongsakon *et al*, 2005 [53] | Cost consequences analysis |
| 54 | Langley-Hawthorne *et al*, 1997 [54] | Partial economic evaluation |
| 55 | Launois *et al*, 1998 [55] | Published before 2005 |
| 56 | Laurier *et al*, 1997 [56] | Published before 2005 |
| 57 | Lecomte *et al*, 2000 [57] | Published before 2005 |
| 58 | Leitao *et al*, 2006 [58] | Partial economic evaluation |
| 59 | Lin *et al*, 2001 [59] | Not modelling study |
| 60 | Lin *et al*, 2015 [60] | Partial economic evaluation |
| 61 | Matheson *et al*, 1994 [61] | Published before 2005 |
| 62 | Mauskopf *et al*, 1999 [62] | Published before 2005 |
| 63 | Mauskopf *et al*, 2002 [63] | Published before 2005 |
| 64 | McCrone *et al*, 2009 [64] | Partial economic evaluation |
| 65 | McCrone *et al*, 2013 [65] | Cost consequences analysis |
| 66 | Mehta *et al*, 2017 [66] | Not modelling study |
| 67 | Mihalopoulos *et al*, 1999 [67] | Not modelling study |
| 68 | Mihalopoulos *et al*, 2004 [68] | Published before 2006 |
| 69 | Mortimer *et al*, 2003 [69] | Published before 2005 |
| 70 | Nemeth *et al*, 2018 [70] | Review paper |
| 71 | Norton *et al*, 2006 [71] | Not economic evaluation |
| 72 | Oh *et al*, 2001 a [72] | Published before 2005 |
| 73 | Oh *et al*, 2001 [73] | Published before 2005 |
| 74 | O'Malley *et al*, 2011 [74] | Partial economic evaluation |
| 75 | Osborn *et al*, 2019 [75] | Not economic evaluation |
| 76 | Palmer *et al*, 1998 [76] | Published before 2005 |
| 77 | Palmer *et al*, 2002 [77] | Published before 2005 |
| 78 | Patel *et al*, 2013 [78] | Not modelling study |
| 79 | Petit *et al*, 2003 [79] | Not modelling study |
| 80 | Perez *et al,* 2015 [80] | Different intervention |
| 81 | Perlis *et al*, 2005 [81] | Different intervention |
| 82 | Phanthunane et al. 2011 [82] | Different intervention |
| 83 | Quintero *et al*, 2016 [83] | Partial economic evaluation |
| 84 | Rajagopalan *et al*, 2013 a [84] | Cost consequences analysis |
| 85 | Rajagopalan *et al*, 2013 b [85] | Cost consequences analysis |
| 86 | Rejon-Parrilla *et al*, 2014 [86] | Different intervention |
| 87 | Richardson *et al*, 2015 [87] | Different population |
| 88 | Rosenheck *et al*, 2016 [88] | Not modelling study |
| 89 | Seghers *et al*, 2015 [89] | Not modelling study |
| 90 | Serretti *et al*, 2009 [90] | Cost consequences analysis |
| 91 | Smith *et al,* 2013 [91] | Different intervention |
| 92 | Tilden *et al*, 2002 [92] | Published before 2005 |
| 93 | Valmaggia *et al*, 2009 [93] | Cost consequences analysis |
| 94 | Vera-Llonch *et al*, 2004 [94] | Cost consequences analysis |
| 95 | Verma *et al*, 2011 [95] | Not economic evaluation |
| 96 | Wang *et al*, 2004 [96] | Published before 2005 |
| 97 | Ward *et al*, 2013 [97] | Cost consequences analysis |
| 98 | Wijnen *et al*, 2019 [98] | Different intervention |
| 99 | Windmeijer *et al*, 2006 [99] | Not modelling study |
| 100 | Winkler *et al*, 2018 [100] | Partial economic evaluation |
| 101 | Yu *et al*, 2009 [101] | Not modelling study |
| 102 | Zala *et al*, 2019 [102] | Different intervention |
| 103 | Zhou *et al*, 2018 [103] | Review paper |
| 104 | Zito *et al*, 1995 [104] | Not economic evaluation |

**Notes:**

1. A study can be excluded from the systematic review for more than one reason. In S3 Table we only reported the primary reason for exclusion for each study.

**References**

1. Alexeyeva IM, J. Earnshaw, S. R. Stauffer, V. L. Gibson, J. P. Ascher-Svanum, H. Ramsey, J. Comparing olanzapine and ziprasidone in the treatment of schizophrenia: a case study in modeling. Journal of Medical Economics. 2001;4:179-92.

2. Almond SOD, O. Cost analysis of the treatment of schizophrenia in the UK: a comparison of olanzapine and haloperidol. Pharmacoeconomics. 1998;13(5 Pt 2):575-88.

3. Almond SOD, O. Cost analysis of the treatment of schizophrenia in the UK. A simulation model comparing olanzapine, risperidone and haloperidol. Pharmacoeconomics. 2000;17(4):383-9.

4. Andrews GS, K. Corry, J. Issakidis, C. Lapsley, H. Cost-effectiveness of current and optimal treatment for schizophrenia. British Journal of Psychiatry. 2003;183:427-35.

5. Annemans LE, I. Smet, A. Jacobs, A. Bergman, G. The impact of treatment with risperidone long-acting injection on the Belgian healthcare system: results from a budget impact model. Acta Clin Belg. 2012;67(2):108-19.

6. Arteaga Duarte CHF, E. Van Gils, C. Guillon, P. The clinical and economic impact of three-monthly long-acting formulation of paliperidone palmitate versus the one-monthly formulation in the treatment of schizophrenia in France: A cost-utility study. L'Encephale. 2019;45(6):459-67. doi:<https://dx.doi.org/10.1016/j.encep.2019.03.001>.

7. Barnett PGS, J. Y. Krystal, J. H. Rosenheck, R. A. C. S. P. Research Group. Cost and cost-effectiveness in a randomized trial of long-acting risperidone for schizophrenia. Journal of Clinical Psychiatry. 2012;73(5):696-702. doi:<http://dx.doi.org/10.4088/JCP.11m07070>.

8. Barnett PGW, W. Jeffers, A. Hall, S. M. Prochaska, J. J. Cost-effectiveness of smoking cessation treatment initiated during psychiatric hospitalization: Analysis from a randomized, controlled trial. Journal of Clinical Psychiatry. 2015;76(10):e1285-e91. doi:<http://dx.doi.org/10.4088/JCP.14m09016>.

9. Basu AB, C. Alphs, L. Projecting the potential effect of using paliperidone palmitate once-monthly and once-every-3-months long-acting injections among medicaid beneficiaries with schizophrenia. Journal of Managed Care and Specialty Pharmacy. 2018;24(8). doi:<http://dx.doi.org/10.18553/jmcp.2018.24.8.759>.

10. Bera RO, S. Zubek, D. Lau, G. Lin, J. Karson, C. Hospitalization resource utilization and costs among Medicaid insured patients with schizophrenia with different treatment durations of long-acting injectable antipsychotic therapy. J Clin Psychopharmacol. 2014;34(1):30-5. doi:<http://dx.doi.org/10.1097/JCP.0b013e3182a6082a>.

11. Bettinger TLS, G. Jones, D. R. Wilson, J. P. Schizophrenia: multi-attribute utility theory approach to selection of atypical antipsychotics. Ann Pharmacother. 2007;41(2):201-7.

12. Bobes JC, Fernando Rejas, Javier Mackell, Joan. Economic consequences of the adverse reactions related with antipsychotics: An economic model comparing tolerability of ziprasidone, olanzapine, risperidone, and haloperidol in Spain. Progress in Neuro-Psychopharmacology & Biological Psychiatry. 2004;28(8):1287-97. doi:<http://dx.doi.org/10.1016/j.pnpbp.2004.06.017>.

13. Bouvy JCK, M. A. Shah, R. R. Schellekens, H. The cost-effectiveness of drug regulation: the example of thorough QT/QTc studies. Clinical Pharmacology and Therapeutics. 2012;91(2):281-8.

14. Byrom BDG, Chris J. Kilpatrick, Andrew T. Influence of antipsychotic profile on cost of treatment of schizophrenia: A decision analysis approach. International Journal of Psychiatry in Clinical Practice. 1998;2(2):129-38. doi:<http://dx.doi.org/10.3109/13651509809115345>.

15. Carswell CW, A. Vanderpyl, J. Robinson, E. Comparative effectiveness of long-acting risperidone in New Zealand: a report of resource utilization and costs in a 12-month mirror-image analysis. Clinical Drug Investigation. 2010;30(11):777-87. doi:<http://dx.doi.org/10.2165/11537680-000000000-00000>.

16. Colombo GLC, M. Di Matteo, S. Rossi, A. An economic evaluation of aripiprazole vs olanzapine adapted to the Italian setting using outcomes of metabolic syndrome and risk for diabetes in patients with schizophrenia. Neuropsychiatric Disease and Treatment. 2008;4(5):967-76.

17. Davies LMD, Michael F. Assessment of costs and benefits of drug therapy for treatment-resistant schizophrenia in the United Kingdom. The British Journal of Psychiatry. 1993;162:38-42. doi:<http://dx.doi.org/10.1192/bjp.162.1.38>.

18. Davies AL, Paul C. Keks, Nicholas A. Catts, Stanley V. Lambert, Tim Schweitzer, Isaac. Risperidone versus haloperidol: II. Cost-effectiveness. Clinical Therapeutics: The International Peer-Reviewed Journal of Drug Therapy. 1998;20(1):196-213. doi:<http://dx.doi.org/10.1016/S0149-2918%2898%2980046-5>.

19. Davies LML, S. Jones, P. B. Barnes, T. R. Gaughran, F. Hayhurst, K. Markwick, A. Lloyd, H. Cost-effectiveness of first- v second-generation antipsychotic drugs: results from a randomised controlled trial in schizophrenia responding poorly to previous therapy. British Journal of Psychiatry. 2007;191:14-22.

20. Davies LMB, T. R. Jones, P. B. Lewis, S. Gaughran, F. Hayhurst, K. Markwick, A. Lloyd, H. A randomized controlled trial of the cost-utility of second-generation antipsychotics in people with psychosis and eligible for clozapine. Value in Health. 2008;11(4):549-62.

21. de Menil VK, M. McDaid, D. Raja, S. Kingori, J. Waruguru, M. Wood, S. K. Mannarath, S. Lund, C. Cost-effectiveness of the Mental Health and Development model for schizophrenia-spectrum and bipolar disorders in rural Kenya. Psychological medicine. 2015;45(13):2747-56. doi:<http://dx.doi.org/10.1017/S0033291715000719>.

22. Dickey BN, S. L. Toward a model for testing the relationship between quality of care and costs. J Ment Health Policy Econ. 2004;7(1):15-21.

23. Druais SD, A. Cognet, M. Godet, A. Lancon, C. Levy, P. Samalin, L. Guillon, P. Comparison of medical and economic benefits of antipsychotics in the treatment of schizophrenia in France. Encephale. 2017;43(4):311-20. doi:<http://dx.doi.org/10.1016/j.encep.2016.02.021>.

24. Duggan AW, J. Knapp, M. Kerwin, R. Modelling the impact of clozapine on suicide in patients with treatment-resistant schizophrenia in the UK. British Journal of Psychiatry. 2003;182:505-8.

25. Edwards NCL, Julie C. Rupnow, Marcia F. Diamond, Ronald J. Cost effectiveness of long-acting risperidone injection versus alternative antipsychotic agents in patients with schizophrenia in the USA. PharmacoEconomics. 2005;23(Suppl 1):75-89. doi:<http://dx.doi.org/10.2165/00019053-200523001-00007>.

26. Edwards NCR, M. F. Pashos, C. L. Botteman, M. F. Diamond, R. J. Cost-effectiveness model of long-acting risperidone in schizophrenia in the US. Pharmacoeconomics. 2005;23(3):299-314.

27. Edwards NCP, J. Meletiche, D. M. Engelhart, L. Thompson, A. K. Sherr, J. Dirani, R. One-year clinical and economic consequences of oral atypical antipsychotics in the treatment of schizophrenia. Current Medical Research and Opinion. 2008;24(12):3341-55.

28. Edwards NCM, E. Doshi, D. Fastenau, J. The threshold rate of oral atypical anti-psychotic adherence at which paliperidone palmitate is cost saving. Journal of Medical Economics. 2012;15(4):623-34. doi:<http://dx.doi.org/10.3111/13696998.2012.667465>.

29. Einarson TRP, H. Zilbershtein, R. Jensen, R. Vicente, C. Piwko, C. Hemels, M. E. Cost-effectiveness analysis of atypical long-acting antipsychotics for treating chronic schizophrenia in Finland.[Erratum appears in J Med Econ. 2013 Nov;16(11):1366]. Journal of Medical Economics. 2013;16(9):1096-105. doi:<http://dx.doi.org/10.3111/13696998.2013.823869>.

30. Emsley RB, F. Cost-effectiveness of an atypical conventional antipsychotic in South Africa: An economic evaluation of quetiapine versus haloperidol in the treatment of patients partially responsive to previous antipsychotics. South African Journal of Psychiatry. 2004;10(3):58-66.

31. Frey SL, Roland Juckel, Georg Stargardt, Tom. Cost-effectiveness of long-acting injectable risperidone versus flupentixol decanoate in the treatment of schizophrenia: A Markov model parameterized using administrative data. The European Journal of Health Economics. 2014;15(2):133-42. doi:<http://dx.doi.org/10.1007/s10198-013-0460-9>.

32. Ganguly RM, L. S. Martin, B. C. Future employability, a new approach to cost-effectiveness analysis of antipsychotic therapy. Schizophrenia Research. 2003;63(1-2):111-9.

33. Girardin FR, Poncet A, Blondon M, Rollason V, Vernaz N, Chalandon Y et al. Monitoring white blood cell count in adult patients with schizophrenia who are taking clozapine: A cost-effectiveness analysis. The Lancet Psychiatry. 1 (1) (pp 55-62), 2014. Date of Publication: 01 Jun 2014.; 2014.

34. Girardin FR, Poncet A, Perrier A, Vernaz N, Pletscher M, C FS et al. Cost-effectiveness of HLA-DQB1/HLA-B pharmacogenetic-guided treatment and blood monitoring in US patients taking clozapine. Pharmacogenomics Journal. 2019;19(2):211-8. doi:<http://dx.doi.org/10.1038/s41397-017-0004-2>.

35. Glazer WME, L. A pharmacoeconomic model of outpatient antipsychotic therapy in "revolving door" schizophrenic patients. Journal of Clinical Psychiatry. 1996;57(8):337-45.

36. Glennie JLCdEdTdlSd, Quebec. Pharmacoeconomic evaluations of clozapine in treatment-resistant schizophrenia and risperidone in chronic schizophrenia - summary Assertive community treatment for people with persistent and particularly severe mental illness. Ottawa Montreal: Canadian Coordinating Office for Health Technology Assessment/Office Canadien de Coordination de l'Evaluation des Technologues de la Sante (CCOHTA) Conseil d'Evaluation des Technologies de la Sante du Quebec (CETS); 1997.

37. Gozlan GL, L. Monfort, A. S. Doz, M. Ortiz, I. Larroumets, P. Lafuma, A. Cost-effectiveness analysis of aripiprazole once-monthly versus paliperidone palmitate once-monthly in the treatment of schizophrenia in France. Encephale. 2018;44(6):496-503. doi:<http://dx.doi.org/10.1016/j.encep.2018.10.001>.

38. Grande AJR, Wagner Silva Faustino, Christine de Miranda, Claudio Torres McDaid, David Fry, Andra de Moraes, Silvia Helena Mendonca de Oliveira, Sandra Maria do Valle Leone de Farias, Joni Marcio de Tarso Coelho Jardim, Paulo King, Derek Silva, Valter Ziebold, Carolina Evans-Lacko, Sara. Effective/cost effective interventions of child mental health problems in low- and middle-income countries (LAMIC): Systematic review. Medicine. 2020;99(1):e18611. doi:<https://dx.doi.org/10.1097/MD.0000000000018611>.

39. Greenhalgh J, Knight C, Hind D, Beverley C, Walters S. Clinical and cost-effectiveness of electroconvulsive therapy for depressive illness, schizophrenia, catatonia and mania: Systematic reviews and economic modelling studies. Health Technology Assessment. 2005;9(9):iii-94.

40. Grieve RS, J. S. Hu, T. W. Bloom, J. R. Evaluating health care programs by combining cost with quality of life measures: a case study comparing capitation and fee for service. Health Serv Res. 2008;43(4):1204-22. doi:<http://dx.doi.org/10.1111/j.1475-6773.2008.00834.x>.

41. Gutierrez-Recacha P, Chisholm D, Haro JM, Salvador-Carulla L, Ayuso-Mateos JL. Cost-effectiveness of different clinical interventions for reducing the burden of schizophrenia in Spain. Acta Psychiatrica Scandinavica. 2006;432:29-38.

42. Haby MMC, R. Mihalopoulos, C. Magnus, A. Sanderson, K. Andrews, G. Vos, T. Assessing cost-effectiveness - Mental health: Introduction to the study and methods. Australian and New Zealand Journal of Psychiatry. 2004;38(8):569-78.

43. Hansen KF, C. Toumi, M. Lancon, C. A pharmacoeconomic evaluation of zuclopenthixol compared with haloperidol and risperidone in the treatment of schizophrenia. European Journal of Health Economics. 2002;3:173-9.

44. Heeg BB, E. Knapp, M. van Aalst, G. Dries, P. J. de Haan, L. van Hout, B. A. Modelling the treated course of schizophrenia: development of a discrete event simulation model. Pharmacoeconomics. 2005;23 Suppl 1:17-33.

45. Henrique ICBdML, Tacio de Melo, Daniela Oliveira Aguiar, Patricia Melo. Economic evaluations on the use of aripiprazole for patients with schizophrenia: A systematic review. Journal of clinical pharmacy and therapeutics. 2020;45(1):1-15. doi:<https://dx.doi.org/10.1111/jcpt.13034>.

46. Janssen BS-K, C. Gaebel, W. Complex intervention in schizophrenia. Results of an integrated care project in German psychiatry and psychotherapy. European Psychiatry. 2011;26.

47. Jin H, McCrone P, MacCabe JH. Stratified medicine in schizophrenia: how accurate would a test of drug response need to be to achieve cost-effective improvements in quality of life? European Journal of Health Economics. 2019;20(9):1425-35. doi:<http://dx.doi.org/10.1007/s10198-019-01108-4>.

48. Johnson-Masotti APP, S. D. Kelly, J. A. Stevenson, L. Y. Cost-effectiveness of an HIV risk reduction intervention for adults with severe mental illness. AIDS Care. 2000;12(3):321-32.

49. Joshi KL, J. Lingohr-Smith, M. Fu, D. J. Estimated medical cost reductions for paliperidone palmitate vs placebo in a randomized, double-blind relapse-prevention trial of patients with schizoaffective disorder. Journal of Medical Economics. 2015;18(8):629-36.

50. Kaaya SE, E. Lapidos-Salaiz, I. Musisi, S. Psaros, C. Wissow, L. Grand Challenges: Improving HIV Treatment Outcomes by Integrating Interventions for Co-Morbid Mental Illness. PLoS Medicine. 2013;10(5).

51. Karki SDB, T. J. Patil, K. Oretega, T. Cost-effectiveness of atypical antipsychotics in severely and persistently mentally III patients with schizophrenia and schizoaffective disorders. Drug Benefit Trends. 2001;13(2):7BH-9BH+11BH-2BH+5BH.

52. Keks NA. Impact of newer antipsychotics on outcomes in schizophrenia. Clinical Therapeutics: The International Peer-Reviewed Journal of Drug Therapy. 1997;19(1):148-58. doi:<http://dx.doi.org/10.1016/S0149-2918%2897%2980082-3>.

53. Kongsakon RL, T. Price, N. Birinyi-Strachan, L. Davey, P. Cost analysis of the treatment of schizophrenia in Thailand: a simulation model comparing olanzapine, risperidone, quetiapine, ziprasidone and haloperidol. J Med Assoc Thai. 2005;88(9):1267-77.

54. Langley-Hawthorne C. Modeling the lifetime costs of treating schizophrenia in Australia. Clinical Therapeutics: The International Peer-Reviewed Journal of Drug Therapy. 1997;19(6):1470-95. doi:<http://dx.doi.org/10.1016/S0149-2918%2897%2980020-3>.

55. Launois RVDS, M. G. Knapp, M. Toumi, M. Cost-effectiveness of sertindole versus olanzapine or haloperidol: A comprehensive model. International Journal of Psychiatry in Clinical Practice. 1998;2(SUPPL. 2):S79-S86.

56. Laurier CK, W. Lachaine, J. Gariepy, L. Tessier, G. Economic evaluation of zuclopenthixol acetate compared with injectable haloperidol in schizophrenic patients with acute psychosis. Clin Ther. 1997;19(2):316-29.

57. Lecomte PDH, M. van Dijk, M. Nuijten, M. Nuyts, G. Persson, U. A 1-year cost-effectiveness model for the treatment of chronic schizophrenia with acute exacerbations in Belgium. Value in Health. 2000;3(1):1-11.

58. Leitao RJF, M. B. Chaves, A. C. Mari, J. J. Cost of schizophrenia: Direct costs and use of resources in the State of Sao Paulo. Revista de Saude Publica. 2006;40(2):304-9.

59. Lin ECY, T. J. Kuo, B. I. Chang, W. Y. Tsai, S. L. A comparison of effectiveness and cost between two models of care for individuals with schizophrenia living in Taiwan. Archives of Psychiatric Nursing. 2001;15(6):272-8.

60. Lin IM, E. Munsell, M. Benson, C. Menzin, J. Economic impact of psychiatric relapse and recidivism among adults with schizophrenia recently released from incarceration: a Markov model analysis. Journal of Medical Economics. 2015;18(3):219-29. doi:<http://dx.doi.org/10.3111/13696998.2014.971161>.

61. Matheson LAC, H. M. McKenna, P. Bosanquet, N. Value for money care for patients with schizophrenia. British Journal of Medical Economics. 1994;7(I):25-34.

62. Mauskopf JAD, Kevin Grainger, David L. Gibson, P. Annual health outcomes and treatment costs for schizophrenia populations. Journal of Clinical Psychiatry. 1999;60(Suppl 19):14-9.

63. Mauskopf JM, M. Gibson, P. J. Grainger, D. L. Estimating the costs and benefits of new drug therapies: Atypical antipsychotic drugs for schizophrenia. Schizophrenia Bulletin. 28 (4) (pp 619-635), 2002. Date of Publication: 2002.; 2002.

64. McCrone PK, M. Dhanasiri, S. Economic impact of services for first-episode psychosis: a decision model approach. Early Interv Psychiatry. 2009;3(4):266-73. doi:<http://dx.doi.org/10.1111/j.1751-7893.2009.00145.x>.

65. McCrone PS, S. P. Knapp, M. Smith, J. Clark, M. Shiers, D. Tiffin, P. A. The economic impact of early intervention in psychosis services for children and adolescents. Early Interv Psychiatry. 2013;7(4):368-73. doi:<http://dx.doi.org/10.1111/eip.12024>.

66. Mehta SG, R. Lang, K. Improving disease management through insights gained from real-world observational data. Value in Health. 2017;20 (5):A343.

67. Mihalopoulos CM, P. D. Carter, R. C. Is phase-specific, community-oriented treatment of early psychosis an economically viable method of improving outcome? Acta Psychiatrica Scandinavica. 1999;100(1):47-55.

68. Mihalopoulos CM, A. Carter, R. Vos, T. Assessing cost-effectiveness in mental health: family interventions for schizophrenia and related conditions. Australian and New Zealand Journal of Psychiatry. 2004;38(7):511-9.

69. Mortimer AW, P. Meddis, D. Impact of side-effects of atypical antipsychotics on non-compliance, relapse and cost. Journal of International Medical Research. 2003;31(3):188-96.

70. Nemeth BF, A. Molnar, A. Bitter, I. Horvath, M. Koczian, K. Gotze, A. Nagy, B. A systematic review of health economic models and utility estimation methods in schizophrenia. Expert Review of Pharmacoeconomics and Outcomes Research. 2018;18(3):267-75. doi:<http://dx.doi.org/10.1080/14737167.2018.1430571>.

71. Norton ECY, J. Domingo, M. E. Morrisey, J. P. Transitions between the public mental health system and jail for persons with severe mental illness: A Markov analysis. Health Economics. 2006;15(7):719-33.

72. Oh PII, M. Addis, A. Lanctot, K. Einarson, T. R. Pharmacoeconomic evaluation of clozapine in treatment-resistant schizophrenia: a cost-utility analysis. Canadian Journal of Clinical Pharmacology. 2001;8(4):199-206.

73. Oh PIL, K. L. Mittmann, N. Iskedjian, M. Einarson, T. R. Cost-utility of risperidone compared with standard conventional antipsychotics in chronic schizophrenia. Journal of Medical Economics. 2001;4(137-156):137-56.

74. O'Malley AJF, R. G. Normand, S. L. T. Estimating cost-offsets of new medications: Use of new antipsychotics and mental health costs for schizophrenia. Statistics in Medicine. 2011;30(16):1971-88.

75. Osborn DB, Alexandra Walters, Kate Atkins, Lou Barnes, Thomas Blackburn, Ruth Craig, Thomas Gilbert, Hazel Gray, Ben Hardoon, Sarah Heinkel, Samira Holt, Richard Hunter, Rachael Johnston, Claire King, Michael Leibowitz, Judy Marston, Louise Michie, Susan Morris, Richard Morris, Steve Nazareth, Irwin Omar, Rumana Petersen, Irene Peveler, Robert Pinfold, Vanessa Stevenson, Fiona Zomer, Ella. Primary care management of cardiovascular risk for people with severe mental illnesses: the Primrose research programme including cluster RCT. 2019. doi:<https://dx.doi.org/10.3310/pgfar07020>.

76. Palmer CSR, D. A. Genduso, L. A. Hamilton, S. H. Brown, R. E. A cost-effectiveness clinical decision analysis model for schizophrenia. Am J Manag Care. 1998;4(3):345-55.

77. Palmer CSB, E. Ruiz-Flores, L. G. Paez-Agraz, F. Revicki, D. A. A cost-effectiveness clinical decision analysis model for treatment of Schizophrenia. Arch Med Res. 2002;33(6):572-80.

78. Patel AM, P. Leese, M. Amaddeo, F. Tansella, M. Kilian, R. Angermeyer, M. Kikkert, M. Schene, A. Knapp, M. Cost-effectiveness of adherence therapy versus health education for people with schizophrenia: Randomised controlled trial in four European countries. Cost Effectiveness and Resource Allocation. 2013;11(1).

79. Petit CM, J. A Bayesian analysis of pharmacoeconomic data from a clinical trial on schizophrenia. Statistics in Medicine. 2003;22(6):1025-39.

80. Perez J, Jin H, Russo DA, Stochl J, Painter M, Shelley G et al. Clinical effectiveness and cost-effectiveness of tailored intensive liaison between primary and secondary care to identify individuals at risk of a first psychotic illness (the LEGs study): a cluster-randomised controlled trial. The Lancet Psychiatry. 2015;2(11):984-93. doi:10.1016/s2215-0366(15)00157-1.

81. Perlis RH, Ganz DA, Avorn J, Schneeweiss S, Glynn RJ, Smoller JW et al. Pharmacogenetic testing in the clinical management of schizophrenia: a decision-analytic model. Journal of Clinical Psychopharmacology. 2005;25(5):427-34.

82. Phanthunane P. Cost-effectiveness of pharmacological and psychosocial interventions for schizophrenia. Cost Effectiveness and Resource Allocation. 2011;9(6):1-9.

83. Quintero JO, I. Gonzalez, B. Cuervo-Arango, I. Garcia, I. Casado, M. A. Cost-Minimisation Analysis of Paliperidone Palmitate Long-Acting Treatment versus Risperidone Long-Acting Treatment for Schizophrenia in Spain. Clinical Drug Investigation. 2016;36(6):479-90.

84. Rajagopalan KH, M. O'Day, K. Meyer, K. Grossman, F. Cost-effectiveness of lurasidone vs aripiprazole among patients with schizophrenia who have previously failed on an atypical antipsychotic: an indirect comparison of outcomes from clinical trial data. Journal of Medical Economics. 2013;16(7):951-61. doi:<http://dx.doi.org/10.3111/13696998.2013.807813>.

85. Rajagopalan KOD, K. Meyer, K. Pikalov, A. Loebel, A. Annual cost of relapses and relapse-related hospitalizations in adults with schizophrenia: results from a 12-month, double-blind, comparative study of lurasidone vs quetiapine extended-release. Journal of Medical Economics. 2013;16(8):987-96. doi:<http://dx.doi.org/10.3111/13696998.2013.809353>.

86. Rejon-Parrilla JC, Nuijten M, Redekop WK, Gaultney JG. Economic evaluation of the use of a pharmacogenetic diagnostic test in schizophrenia. Health Policy and Technology. 3 (4) (pp 314-324), 2014. Date of Publication: 01 Dec 2014.; 2014.

87. Richardson RT, D. Perry, A. Ali, S. Duffy, S. Gabe, R. Gilbody, S. Glanville, J. Hewitt, C. Manea, L. Palmer, S. Wright, B. McMillan, D. Screening for psychological and mental health difficulties in young people who offend: a systematic review and decision model. Health Technol Assess. 2015;19(1):1-128. doi:<http://dx.doi.org/10.3310/hta19010>.

88. Rosenheck RL, D. Sint, K. Lin, H. Robinson, D. G. Schooler, N. R. Mueser, K. T. Penn, D. L. Addington, J. Brunette, M. F. Correll, C. U. Estroff, S. E. Marcy, P. Robinson, J. Severe, J. Rupp, A. Schoenbaum, M. Kane, J. M. Cost-effectiveness of comprehensive, integrated care for first episode psychosis in the nimh raise early treatment program. Schizophrenia Bulletin. 2016;42(4):896-906. doi:<http://dx.doi.org/10.1093/schbul/sbv224>.

89. Seghers ACS, K. H. Chio, M. T. W. Chia, E. Ng, S. K. Tang, M. B. Y. A prospective study on the use of teledermatology in psychiatric patients with chronic skin diseases. Australasian Journal of Dermatology. 2015;56(3):170-4. doi:<http://dx.doi.org/10.1111/ajd.12297>.

90. Serretti AM, L. Bajo, E. Cevenini, N. Papili, P. Mori, E. Bigelli, M. Berardi, D. The socio-economical burden of schizophrenia: a simulation of cost-offset of early intervention program in Italy. Eur Psychiatry. 2009;24(1):11-6. doi:<http://dx.doi.org/10.1016/j.eurpsy.2008.07.009>.

91. Smith KJ, Baik SH, Reynolds CF, 3rd, Rollman BL, Zhang Y. Cost-effectiveness of Medicare drug plans in schizophrenia and bipolar disorder. American Journal of Managed Care. 2013;19(2).

92. Tilden DA, M. Meddis, D. Burns, T. An economic assessment of quetiapine and haloperidol in patients with schizophrenia only partially responsive to conventional antipsychotics. Clin Ther. 2002;24(10):1648-67.

93. Valmaggia LM, P. Knapp, M. Woolley, J. Broome, M. Tabraham, P. Johns, L. Prescott, C. Bramon, E. Lappin, J. Power, P. McGuire, P. Economic impact of early intervention in people at high risk of psychosis. Psychological Medicine. 2009;39(10):1617-26. doi:<http://dx.doi.org/10.1017/S0033291709005613>.

94. Vera-Llonch MD, Thomas E. Richardson, Erin Rupnow, Marcia Grogg, Amy Oster, Gerry. Outcomes and Costs of Risperidone versus Olanzapine in Patients with Chronic Schizophrenia or Schizoaffective Disorders: A Markov Model. Value in Health. 2004;7(5):569-84. doi:<http://dx.doi.org/10.1111/j.1524-4733.2004.75008.x>.

95. Verma SC, L. L. Chee, K. S. Chen, H. Chin, S. A. Chong, S. A. Chua, W. Fones, C. Fung, D. Khoo, C. L. Kwek, S. K. D. Ling, J. Poh, P. Sim, K. Tan, B. L. Tan, C. Tan, C. H. Tan, L. L. Tay, W. K. Ministry of Health Clinical Practice Guidelines: Schizophrenia. Singapore Medical Journal. 2011;52(7):521-6.

96. Wang PSG, D. A. Benner, J. S. Glynn, R. J. Avorn, J. Should clozapine continue to be restricted to third-line status for schizophrenia?: a decision-analytic model. J Ment Health Policy Econ. 2004;7(2):77-85.

97. Ward AQ, P. Abouzaid, S. Haber, N. Ahmed, S. Kim, E. Cardiometabolic consequences of therapy for chronic schizophrenia using second-generation antipsychotic agents in a medicaid population: clinical and economic evaluation. P T. 2013;38(2):109-15.

98. Wijnen BFM, Thielen FW, Konings S, Feenstra T, Van Der Gaag M, Veling W et al. Designing and Testing of a Health-Economic Markov Model for Prevention and Treatment of Early Psychosis. Expert review of pharmacoeconomics & outcomes research. 2019:1-11. doi:<https://dx.doi.org/10.1080/14737167.2019.1632194>.

99. Windmeijer FK, S. Knapp, M. Brown, J. Haro, J. M. Methodological approach for assessing the cost-effectiveness of treatments using longitudinal observational data: the SOHO study. Int J Technol Assess Health Care. 2006;22(4):460-8.

100. Winkler PB, Hana Marie Kondratova, Lucie Knapp, Martin Arteel, Paul Boyer, Patrice Galderisi, Silvana Karkkainen, Hikka Ieven, Aagje Mohr, Pavel Wasserman, Danuta Park, A. La Tinelli, Michella Gaebel, Wolfgang. Value of schizophrenia treatment II: Decision modelling for developing early detection and early intervention services in the Czech Republic. European psychiatry : the journal of the Association of European Psychiatrists. 2018;53:116-22. doi:<https://dx.doi.org/10.1016/j.eurpsy.2018.06.008>.

101. Yu APA, P. Ben-Hamadi, R. Birnbaum, H. Stensland, M. D. Philips, G. Resource utilization and costs of schizophrenia patients treated with olanzapine versus quetiapine in a Medicaid population. Value in Health. 2009;12(5):708-15. doi:<http://dx.doi.org/10.1111/j.1524-4733.2008.00498.x>.

102. Zala D, Brabban A, Stirzaker A, Kartha MR, McCrone P. The Cost-Effectiveness of the Improving Access to Psychological Therapies (IAPT) Programme in Severe Mental Illness: A Decision Analytical Model Using Routine Data. Community mental health journal. 2019;55(5):873-83. doi:<https://dx.doi.org/10.1007/s10597-019-00390-z>.

103. Zhou JM, Aurelie Toumi, Mondher. Systematic review of pharmacoeconomic models for schizophrenia. Journal of market access & health policy. 2018;6(1):1508272. doi:<https://dx.doi.org/10.1080/20016689.2018.1508272>.

104. Zito JMP, George. Pharmaceutical decisionmaking: Pharmacoepidemiology or pharmacoeconomics: Who's in the driver's seat? Psychopharmacology Bulletin. 1995;31(4):735-44.
