# Supplementary material for: Systematic review of the methods of health economic models assessing antipsychotic medication for schizophrenia
Source: PLoS One. 2020 Jul 10;15(7):e0234996. doi: 10.1371/journal.pone.0234996 (PMC7351140; doi:10.1371/journal.pone.0234996)
Supplement: S5 Table — (DOCX) [file pone.0234996.s006.docx]

**S5 Table. Number and type of antipsychotic medications covered by included studies**

|  | **Included studies**  n (%) |
| --- | --- |
| **Number of antipsychotic medications covered by each study** |  |
| 1^1^ | 4 (6.7) |
| 2 | 17 (28.3) |
| 3-4 | 22 (38.3) |
| 5-10 | 15 (25.0) |
| 11 | 1 (1.7) |
| **Frequency of antipsychotic medications assessed by included studies** | |
| Risperidone | 43 (71.7) |
| Olanzapine | 42 (70.0) |
| Paliperidone | 22 (36.7) |
| Aripiprazole | 17 (28.3) |
| Haloperidol | 11 (18.3) |
| Quetiapine | 11 (18.3) |
| Ziprasidone | 9 (15.0) |
| Amisulpride | 4 (6.7) |
| Chlorpromazine | 3 (5.0) |
| Clozapine | 3 (5.0) |
| Cariprazine | 2 (3.3) |
| Lurasidone | 2 (3.3) |
| Asenapine | 1 (1.7) |
| Brexpiprazole | 1 (1.7) |
| Sertindole | 1 (1.7) |
| Sulpiride | 1 (1.7) |
| Trifluoperazine | 1 (1.7) |
| Zotepine | 1 (1.7) |
| Zuclopenthixol | 1 (1.7) |
| **Type of antipsychotic medications (by administration route) covered by included studies** | |
| Oral antipsychotics only | 35 (58.3) |
| Long-acting injectable (LAI) antipsychotics only | 9 (15.0) |
| Both oral and LAI antipsychotics | 16 (26.7%) |

**Notes:**

1. Of the four studies which only assessed one antipsychotic medication, two studies compared one antipsychotic medication with placebo [1, 2], one study compared the same antipsychotic with different compliance level [3], and one study compared branded antipsychotic with generic antipsychotic [4].

**References**

1. Bernardo M, Ramon Azanza J, Rubio-Terres C, Rejas J. Cost-effectiveness analysis of schizophrenia relapse prevention : an economic evaluation of the ZEUS (Ziprasidone-Extended-Use-In-Schizophrenia) study in Spain. Clinical Drug Investigation. 2006;26(8):447-57. PubMed PMID: 17163277.

2. Pribylova L, Kolek M, Vesela S, Duba J, Slesinger J, Doleckova J. De novo cost-utility analysis of oral paliperidone in the treatment of schizoaffective disorder. Journal of Psychiatric Research. 2015;70:33-7. doi: <http://dx.doi.org/10.1016/j.jpsychires.2015.08.013>. PubMed PMID: 606358965.

3. Damen J, Thuresson PO, Heeg B, Lothgren M. A pharmacoeconomic analysis of compliance gains on antipsychotic medications. Appl Health Econ Health Policy. 2008;6(4):189-97. doi: <http://dx.doi.org/10.2165/00148365-200806040-00002>. PubMed PMID: 19382819.

4. Treur M, Heeg B, Moller HJ, Schmeding A, van Hout B. A pharmaco-economic analysis of patients with schizophrenia switching to generic risperidone involving a possible compliance loss. BMC Health Services Research. 2009;9:32. doi: <http://dx.doi.org/10.1186/1472-6963-9-32>. PubMed PMID: 19226465; PubMed Central PMCID: PMCPMC2652458.
