# Supplementary material for: Systematic review of the methods of health economic models assessing antipsychotic medication for schizophrenia
Source: PLoS One. 2020 Jul 10;15(7):e0234996. doi: 10.1371/journal.pone.0234996 (PMC7351140; doi:10.1371/journal.pone.0234996)
Supplement: S6 Table — (DOCX) [file pone.0234996.s007.docx]

**S6 Table: Performance of included studies assessed by Section 2 of the NICE checklist**

| **Reference** | **Question 1**  (Model structure^1^) | **Question 2**  (Time horizon^1^) | **Question 3**  (Important outcomes included?^3^) | **Question 4**  (Baseline health outcome data^4^) | **Question 5** (Relative treatment effects data^5^) | **Question 6** (Important costs included?^6^) | **Question 7**  (Resource use data^7^) | **Question 8**  (Unit cost data8) | **Question 9** (Incremental analysis presented?^9^) | **Question 10**  (Sensitivity analysis^10^) | **Question 11**  (No potential conflict of interest? ^11^) | **Overall assessment** |
| --- | --- | --- | --- | --- | --- | --- | --- | --- | --- | --- | --- | --- |
| Aigbogun *et al. (1)* | Yes | Partly | Yes | Partly | Partly | Yes | Yes | Partly | Yes | Yes | Yes | Very serious limitations |
| Anh *et al.* *(2)* | Partly | Yes | Partly | Partly | Yes | Partly | Partly | Yes | Yes | Partly | No | Very serious limitations |
| Ascher-Svanum *et al.(3)* | Yes | Partly | Yes | Yes | Yes | Yes | Yes | Partly | Yes | Yes | Yes | Potentially serious limitations |
| Beard *et al.(4)* | Partly | Partly | Partly | No | Yes | Partly | No | Partly | Yes | Partly | Yes | Very serious limitations |
| Bernardo *et al.(5)* | Not clear | Partly | Not clear | Partly | Yes | Not clear | Partly | Yes | Yes | Partly | Yes | Very serious limitations |
| Bounthavong *et al.(6)* | Yes | No | Partly | Partly | Yes | Partly | Partly | Not clear | Yes | Partly | No | Very serious limitations |
| Chisholm *et al.* *2012* *(7)* | Partly | Yes | Partly | Yes | Yes | Partly | Partly | Partly | Yes | Partly | No | Very serious limitations |
| Chisholm *et al.* *2008 (8)* | Partly | Yes | Partly | Yes | Yes | Partly | Not clear | Not clear | Yes | Partly | No | Very serious limitations |
| Chue *et al.(9)* | Yes | Partly | No | Not clear | Not clear | No | No | Yes | Yes | Partly | Yes | Very serious limitations |
| Citrome *et al.(10)* | Yes | Partly | Yes | Partly | No | Yes | Partly | Partly | Yes | Partly | Yes | Very serious limitations |
| Damen *et al.(11)* | Partly | Partly | No | Not clear | No | No | Not clear | Yes | Yes | Partly | Yes | Very serious limitations |
| Davies *et al.(12)* | Yes | Partly | Partly | Partly | Yes | Yes | No | Yes | Yes | Yes | Yes | Very serious limitations |
| De Graeve *et al.(13)* | Partly | Partly | Partly | No | No | Partly | Yes | Yes | Yes | Partly | Yes | Very serious limitations |
| Dilla *et al.(14)* | Yes | Partly | No | Partly | Yes | No | Partly | Yes | Yes | Yes | Yes | Very serious limitations |
| Druais *et al.(15)* | Yes | Partly | Yes | Partly | No | Yes | Partly | Partly | Yes | Yes | Yes | Very serious limitations |
| Einarson *et al.(16)* | Yes | Partly | No | Partly | Partly | No | Not clear | Yes | Yes | Yes | Yes | Very serious limitations |
| Einarson *et al.(17)* | Yes | Partly | No | Partly | Partly | No | Not clear | Yes | Yes | Yes | Yes | Very serious limitations |
| Einarson *et al.(18)* | Yes | Partly | No | Partly | Partly | No | Partly | Yes | Yes | Yes | Yes | Very serious limitations |
| Einarson *et al.(19)* | Yes | Partly | No | Partly | Partly | No | Not clear | Yes | Yes | Yes | Yes | Very serious limitations |
| Einarson *et al.(20)* | Yes | Partly | No | Partly | No | No | Yes | Yes | Yes | Yes | Yes | Very serious limitations |
| Einarson *et al.(21)* | Yes | Partly | No | Partly | No | No | Not clear | Yes | Yes | Yes | Yes | Very serious limitations |
| Einarson *et al.(22)* | Yes | Partly | No | Partly | Yes | No | Partly | Yes | Yes | Yes | Yes | Very serious limitations |
| Einarson *et al.(23)* | Yes | Partly | No | Partly | No | No | Not clear | Partly | Yes | Yes | Yes | Very serious limitations |
| Furiak *et al.*(24) | Yes | Partly | Yes | Yes | Partly | Yes | Partly | Yes | Yes | Yes | Yes | Very serious limitations |
| Furiak *et al.(25)* | Yes | Partly | Yes | Yes | Partly | Yes | Partly | Partly | Yes | Yes | Yes | Very serious limitations |
| Garcia-Ruiz *et al.(26)* | Yes | Partly | Yes | Partly | Yes | Yes | Yes | Yes | Yes | Partly | Yes | Very serious limitations |
| Geitona *et al.(27)* | Yes | Partly | Partly | Partly | Yes | Yes | No | Yes | Yes | Partly | Yes | Very serious limitations |
| Girardin *et al.* (28) | No | Partly | No | Not clear | Not clear | No | Partly | Partly | Yes | Yes | No | Very serious limitations |
| Heeg *et al.(29)* | Yes | Partly | Yes | Partly | Yes | No | Not clear | Yes | Yes | Partly | Yes | Very serious limitations |
| Heeg *et al.(30)* | Yes | Partly | Yes | Partly | Yes | Yes | Partly | Yes | Yes | Yes | Yes | Very serious limitations |
| Hensen *et al.(31)* | Yes | Partly | Yes | Not clear | Yes | No | Yes | Yes | Yes | Yes | Yes | Very serious limitations |
| Jukic *et al.(32)* | Yes | Partly | No | Yes | No | No | Not clear | Yes | Yes | Yes | Yes | Very serious limitations |
| Kasteng *et al.(33)* | No | Yes | No | Partly | Yes | No | Partly | Yes | Yes | Yes | Yes | Very serious limitations |
| Kim *et al.(34)* | No | Partly | No | Partly | Yes | No | Yes | Yes | Yes | Yes | Yes | Very serious limitations |
| Kim *et al.(35)* | Yes | Partly | Partly | Partly | Yes | Partly | Partly | Yes | Yes | Yes | Yes | Very serious limitations |
| Lachaine *et al.(36)* | Partly | Partly | Partly | Partly | Yes | No | Yes | Yes | Yes | Yes | Yes | Very serious limitations |
| Laux *et al.(37)* | Yes | Partly | Partly | Partly | Yes | No | Partly | Partly | Yes | Partly | Yes | Very serious limitations |
| Lin *et al.(38)* | Yes | Yes | Yes | Partly | Yes | Yes | Yes | Yes | Yes | Yes | No | Minor limitations |
| Lindner *et al.(39)* | Partly | Partly | No | Partly | Yes | No | Yes | Yes | Yes | Partly | No | Very serious limitations |
| Lindstrom *et al.(40)* | Yes | Partly | Yes | Partly | Partly | Yes | Yes | Yes | Yes | Yes | Yes | Very serious limitations |
| Lubinga *et al.(41)* | Yes | Yes | Yes | Partly | Partly | Yes | Partly | Partly | Yes | Yes | No | Very serious limitations |
| Magnus *et al.(42)* | No | Yes | No | Yes | Yes | No | Yes | Yes | Yes | Yes | No | Very serious limitations |
| McIntyre *et al.(43)* | No | Partly | Yes | Partly | Yes | Yes | Yes | Yes | Yes | Yes | Yes | Very serious limitations |
| Mehnert *et al.(44)* | Yes | Partly | Yes | Partly | Yes | Yes | Partly | Yes | Yes | Yes | Yes | Very serious limitations |
| Mould-Quevedo *et al.(45)* | Yes | Partly | Yes | No | No | Not clear | No | Yes | Yes | Partly | Yes | Very serious limitations |
| NCCMH *et al.(46)* | Yes | Yes | Yes | Yes | Yes | Yes | Yes | Yes | Yes | Yes | No | Minor limitations |
| Németh *et al.* (47) | Partly | Partly | Partly | Partly | Yes | Partly | Partly | Yes | Yes | Yes | Yes | Very serious limitations |
| Nuhoho *et al.(48)* | Partly | Partly | No | Partly | No | No | No | Yes | Yes | Yes | Yes | Very serious limitations |
| Obradovic *et al.(49)* | Partly | Partly | Partly | No | No | Partly | No | Yes | Yes | Partly | No | Very serious limitations |
| Park *et al.(50)* | Yes | Partly | Yes | Partly | Yes | Yes | Yes | Yes | Yes | Yes | No | Potentially serious limitations |
| Pribylova *et al.(51)* | No | No | No | Partly | Yes | No | No | Partly | Yes | Yes | Yes | Very serious limitations |
| Rajagopalan *et al.(52)* | Not clear | Partly | Yes | Partly | Partly | Yes | Partly | Yes | Yes | Yes | Yes | Very serious limitations |
| Tempest *et al.(53)* | Yes | Partly | Yes | Partly | Yes | Yes | Yes | Yes | Yes | Yes | Yes | Potentially serious limitations |
| Thavornwattanayong *et al.* (54) | Yes | Yes | Yes | Partly | Partly | Yes | Partly | Yes | Yes | Yes | No | Potentially serious limitations |
| Treur *et al.(55)* | Yes | Partly | Yes | Partly | Yes | Yes | No | Yes | Yes | Yes | Yes | Very serious limitations |
| Treur *et al.(56)* | Not clear | Partly | Yes | Not clear | No | Not clear | Not clear | Not reported | Yes | Partly | Yes | Very serious limitations |
| Yang *et al.(57)* | Partly | Partly | Partly | No | No | Partly | No | Yes | Yes | Partly | Yes | Very serious limitations |
| Yang *et al.(58)* | Partly | Partly | Partly | Partly | Yes | Partly | No | Partly | Yes | Partly | Yes | Very serious limitations |
| Zhao *et al.* (59) | Yes | Partly | Yes | Partly | Partly | Yes | No | Yes | Yes | Yes | Yes | Very serious limitations |
| Zeidler *et al.(60)* | Yes | Partly | Yes | Partly | Partly | Partly | Partly | Partly | Yes | Yes | Yes | Very serious limitations |

**Notes:**

1: Does the model structure adequately reflect the nature of the health condition under evaluation?

2: Is the time horizon sufficiently long to reflect all important differences in costs and outcomes?

3: Are all important and relevant health outcomes included?

4: Are the estimates of baseline health outcomes from the best available source?

5: Are the estimates of relative treatment effects from the best available source?

6: Are all important and relevant costs included?

7: Are the estimates of resource use from the best available source?

8: Are the unit costs of resources from the best available source?

9: Is an appropriate incremental analysis presented or can it be calculated from the data?

10: Are all important parameters whose values are uncertain subjected to appropriate sensitivity analysis?

11: Is there no potential conflict of interest?

**References**

1. Aigbogun MS, Liu S, Kamat SA, Sapin C, Duhig AM, Citrome L. Relapse prevention: A cost-effectiveness analysis of brexpiprazole treatment in adult patients with schizophrenia in the USA. ClinicoEconomics and Outcomes Research. 2018;10:443-56.

2. Anh NQ, Linh BN, Ha NT, Phanthunane P, Huong NT. Schizophrenia interventions in Vietnam: Primary results from a cost-effectiveness study. Global Public Health. 2015;10:S21-S39.

3. Ascher-Svanum H, Furiak NM, Lawson AH, Klein TM, Smolen LJ, Conley RR, et al. Cost-effectiveness of several atypical antipsychotics in orally disintegrating tablets compared with standard oral tablets in the treatment of schizophrenia in the United States. Journal of Medical Economics. 2012;15(3):531-47.

4. Beard AM, Maciver F, Clouth J, Ruther E. A decision model to compare health care costs of olanzapine and risperidone treatment of schizophrenia in Germany. European Journal of Health Economics. 2006;7:165-72.

5. Bernardo M, Ramon Azanza J, Rubio-Terres C, Rejas J. Cost-effectiveness analysis of schizophrenia relapse prevention : an economic evaluation of the ZEUS (Ziprasidone-Extended-Use-In-Schizophrenia) study in Spain. Clinical Drug Investigation. 2006;26(8):447-57.

6. Bounthavong M, Okamoto MP. Decision analysis model evaluating the cost-effectiveness of risperidone, olanzapine and haloperidol in the treatment of schizophrenia. Journal of Evaluation in Clinical Practice. 2007;13(3):453-60.

7. Chisholm D, Saxena S. Cost effectiveness of strategies to combat neuropsychiatric conditions in sub-Saharan Africa and South East Asia: mathematical modelling study. Bmj. 2012;344.

8. Chisholm D, Gureje O, Saldivia S, Calderon MV, Wickremasinghe R, Mendis N, et al. Schizophrenia treatment in the developing world: An interregional and multinational cost-effectiveness analysis: Bulletin of the World Health Organization. 86 (7) (pp 542-551), 2008. Date of Publication: July 2008.; 2008.

9. Chue P, Heeg BM, Buskens E, van Hout BA. Modelling the impact of compliance on the costs and effects of long-acting risperidone in Canada. PharmacoEconomics. 2005;23(Suppl 1):62-74.

10. Citrome L, Kamat SA, Sapin C, Baker RA, Eramo A, Ortendahl J, et al. Cost-effectiveness of aripiprazole once-monthly compared with paliperidone palmitate once-monthly injectable for the treatment of schizophrenia in the United States. Journal of Medical Economics. 2014;17(8):567-76.

11. Damen J, Thuresson PO, Heeg B, Lothgren M. A pharmacoeconomic analysis of compliance gains on antipsychotic medications. Appl Health Econ Health Policy. 2008;6(4):189-97.

12. Davies A, Vardeva K, Loze JY, L'Italien G J, Sennfalt K, Baardewijk M. Cost-effectiveness of atypical antipsychotics for the management of schizophrenia in the UK. Curr Med Res Opin. 2008;24(11):3275-85.

13. De Graeve D, Smet A, Mehnert, Caleo S, Miadi-Fargier H, Mosqueda GJ, et al. Long-acting risperidone compared with oral olanzapine and haloperidol depot in schizophrenia: a Belgian cost-effectiveness analysis. PharmacoEconomics. 2005;23(Supplement 1):35-47.

14. Dilla T, Moller J, O'Donohoe P, Alvarez M, Sacristan JA, Happich M, et al. Long-acting olanzapine versus long-acting risperidone for schizophrenia in Spain - a cost-effectiveness comparison. BMC Psychiatry. 2014;14(1):298.

15. Druais S, Doutriaux A, Cognet M, Godet A, Lancon C, Levy P, et al. Cost Effectiveness of Paliperidone Long-Acting Injectable Versus Other Antipsychotics for the Maintenance Treatment of Schizophrenia in France. PharmacoEconomics. 2016;34(4):363-91.

16. Einarson TR, Bereza BG, Tedouri F, Van Impe K, Denee TR, Dries PJT. Cost-effectiveness of 3-month paliperidone therapy for chronic schizophrenia in the Netherlands. Journal of Medical Economics. 2017;20(11):1187-99.

17. Einarson TR, Maia-Lopes S, Goswami P, Bereza BG, Van Impe K. Economic analysis of paliperidone long-acting injectable for chronic schizophrenia in Portugal. Journal of Medical Economics. 2016;19(9):913-21.

18. Einarson TR, Pudas H, Goswami P, Van Impe K, Bereza BG. Pharmacoeconomics of long-Acting atypical antipsychotics for acutely relapsed chronic schizophrenia in Finland. Journal of Medical Economics. 2016;19(2):111-20.

19. Einarson TR, Vicente C, Zilbershtein R, Piwko C, Bo CN, Pudas H, et al. Pharmacoeconomics of depot antipsychotics for treating chronic schizophrenia in Sweden. Nordic Journal of Psychiatry. 2014;68(6):416-27.

20. Einarson TR, Pudas H, Zilbershtein R, Jensen R, Vicente C, Piwko C, et al. Cost-effectiveness analysis of atypical long-acting antipsychotics for treating chronic schizophrenia in Finland. Journal of Medical Economics. 2013;16(9):1096-105.

21. Einarson TR, Vicente C, Zilbershtein R, Piwko C, Bo CN, Pudas H, et al. Pharmacoeconomic analysis of paliperidone palmitate versus olanzapine pamoate for chronic schizophrenia in Norway. Acta Neuropsychiatrica. 2013;25(2):85-94.

22. Einarson TR, Zilbershtein R, Skoupa J, Vesela S, Garg M, Hemels ME. Economic and clinical comparison of atypical depot antipsychotic drugs for treatment of chronic schizophrenia in the Czech Republic. Journal of Medical Economics. 2013;16(9):1089-95.

23. Einarson TR, Geitona M, Chaidemenos A, Karpouza V, Mougiakos T, Paterakis P, et al. Pharmacoeconomic analysis of paliperidone palmitate for treating schizophrenia in Greece. Annals of General Psychiatry. 2012;11(1):18.

24. Furiak NM, Ascher-Svanum H, Klein RW, Smolen LJ, Lawson AH, Montgomery W, et al. Cost-effectiveness of olanzapine long-acting injection in the treatment of patients with schizophrenia in the United States: a micro-simulation economic decision model. Curr Med Res Opin. 2011;27(4):713-30.

25. Furiak NM, Ascher-Svanum H, Klein RW, Smolen LJ, Lawson AH, Conley RR, et al. Cost-effectiveness model comparing olanzapine and other oral atypical antipsychotics in the treatment of schizophrenia in the United States. Cost Eff Resour Alloc. 2009;7:4.

26. Garcia-Ruiz AJ, Perez-Costillas L, Montesinos AC, Alcalde J, Oyaguez I, Casado MA. Cost-effectiveness analysis of antipsychotics in reducing schizophrenia relapses. Health Economics Review. 2012;2(1):8.

27. Geitona M, Kousoulakou H, Ollandezos M, Athanasakis K, Papanicolaou S, Kyriopoulos I. Costs and effects of paliperidone extended release compared with alternative oral antipsychotic agents in patients with schizophrenia in Greece: a cost effectiveness study. Annals of General Psychiatry. 2008;7:16.

28. Girardin FR, Poncet A, Perrier A, Vernaz N, Pletscher M, C FS, et al. Cost-effectiveness of HLA-DQB1/HLA-B pharmacogenetic-guided treatment and blood monitoring in US patients taking clozapine. Pharmacogenomics Journal. 2019;19(2):211-8.

29. Heeg B, Antunes J, Figueira M, Jara J, Teixeira J, Palha A, et al. Cost-effectiveness and budget impact of long-acting risperidone in Portugal: A modeling exercise. Current Medical Research and Opinion. 2008;24(2):349-58.

30. Heeg B, Buskens E, Botteman M, Caleo S, Ingham M, Damen J, et al. The cost-effectiveness of atypicals in the UK. Value in Health. 2008;11(7):1007-21.

31. Hensen M, Heeg B, Lothgren M, van Hout B. Cost effectiveness of long-acting risperidone in Sweden. Appl Health Econ Health Policy. 2010;8(5):327-41.

32. Jukic V, Jakovljevic M, Filipcic I, Herceg M, Silic A, Tomljanovic T, et al. Cost-utility analysis of depot atypical antipsychotics for chronic schizophrenia in Croatia2013. 181-8 p.

33. Kasteng F, Eriksson J, Sennfalt K, Lindgren P. Metabolic effects and cost-effectiveness of aripiprazole versus olanzapine in schizophrenia and bipolar disorder. Acta Psychiatrica Scandinavica. 2011;124(3):214-25.

34. Kim BR, Lee TJ, Lee HJ, Park BH, Yang BM. Cost-effectiveness of sertindole among atypical antipsychotics in the treatment of schizophrenia in South Korea. Value in Health Regional Issues. 2012;1(1):59-65.

35. Kim K, Aas E. Cost-effectiveness analysis of olanzapine and risperidone in Norway. J Ment Health Policy Econ. 2011;14(3):125-35.

36. Lachaine J, Beauchemin C, Mathurin K, Gilbert D, Beillat M. Cost-effectiveness of asenapine in the treatment of schizophrenia in Canada. Journal of Medical Economics. 2014;17(4):296-304.

37. Laux G, Heeg B, van Hout BA, Mehnert A. Costs and effects of long-acting risperidone compared with oral atypical and conventional depot formulations in Germany. Pharmacoeconomics. 2005;23 Suppl 1:49-61.

38. Lin L, Zhao YJ, Zhou HJ, Khoo AL, Teng M, Soh LB, et al. Comparative cost-effectiveness of 11 oral antipsychotics for relapse prevention in schizophrenia within Singapore using effectiveness estimates from a network meta-analysis. International Clinical Psychopharmacology. 2016;31(2):84-92.

39. Lindner LM, Marasciulo AC, Farias MR, Grohs GE. Economic evaluation of antipsychotic drugs for schizophrenia treatment within the Brazilian Healthcare System. Revista de Saude Publica. 2009;43 Suppl 1:62-9.

40. Lindstrom E, Eberhard J, Fors BM, Hansen K, Sapin C. A pharmacoeconomic analysis of sertindole in the treatment of schizophrenia in Sweden. Nordic Journal of Psychiatry. 2011;65(6):403-13.

41. Lubinga SJ, Mutamba BB, Nganizi A, Babigumira JB. A cost-effectiveness analysis of antipsychotics for treatment of schizophrenia in Uganda. Value in Health. 2015;18 (3):A121.

42. Magnus A, Carr V, Mihalopoulos C, Carter R, Vos T. Assessing cost-effectiveness of drug interventions for schizophrenia. Australian and New Zealand Journal of Psychiatry. 2005;39(1-2):44-54.

43. McIntyre RS, Cragin L, Sorensen S, Naci H, Baker T, Roussy J-P. Comparison of the metabolic and economic consequences of long-term treatment of schizophrenia using ziprasidone, olanzapine, quetiapine and risperidone in Canada: A cost-effectiveness analysis. Journal of Evaluation in Clinical Practice. 2010;16(4):744-55.

44. Mehnert A, Nicholl D, Pudas H, Martin M, McGuire A. Cost effectiveness of paliperidone palmitate versus risperidone long-acting injectable and olanzapine pamoate for the treatment of patients with schizophrenia in Sweden. Journal of Medical Economics. 2012;15(5):844-61.

45. Mould-Quevedo J, Contreras-Hernandez I, Verduzco W, Mejia-Arangure JM, Garduno-Espinosa J. Cost-effectiveness simulation analysis of schizophrenia at the Instituto Mexicano del Seguro Social: Assessment of typical and atypical antipsychotics. Rev. 2009;2(3):108-18.

46. National Collaborating Centre for Mental Health. Psychosis and schizophrenia in adults: prevention and management. NICE guideline (CG178).2014.

47. Nemeth B, Bendes R, Nagy B, Gotze A, Koczian K, Horvath M, et al. Cost-utility analysis of cariprazine compared to risperidone among patients with negative symptoms of schizophrenia. Health Policy and Technology. 2019;8(1):84-91.

48. Nuhoho S, Saad A, Saumell G, Ribes D, El Khoury AC. Economic evaluation of paliperidone palmitate once monthly for treating chronic schizophrenia patients in the United Arab Emirates. Current Medical Research and Opinion. 2018;34(4):601-11.

49. Obradovic M, Mrhar A, Kos M. Cost-effectiveness of antipsychotics for outpatients with chronic schizophrenia. International Journal of Clinical Practice. 2007;61(12):1979-88.

50. Park T, Kuntz KM. Cost-effectiveness of second-generation antipsychotics for the treatment of schizophrenia. Value in Health. 2014;17(4):310-9.

51. Pribylova L, Kolek M, Vesela S, Duba J, Slesinger J, Doleckova J. De novo cost-utility analysis of oral paliperidone in the treatment of schizoaffective disorder. Journal of Psychiatric Research. 2015;70:33-7.

52. Rajagopalan K, Trueman D, Crowe L, Squirrell D, Loebel A. Cost-Utility Analysis of Lurasidone Versus Aripiprazole in Adults with Schizophrenia. PharmacoEconomics. 2016;34(7):709-21.

53. Tempest M, Sapin C, Beillat M, Robinson P, Treur M. Cost-effectiveness analysis of aripiprazole once-monthly for the treatment of schizophrenia in the UK. Journal of Mental Health Policy and Economics. 2015;18(4):185-200.

54. Thavornwattanayong W, Lertsirimunkong J, Thongkerd N, Pitakthanin N, Wettayanon P, Pongjakpanit H. Cost-effectiveness analysis of aripiprazole compared with risperidone in the treatment of acute schizophrenia patients in Thailand. Thai Journal of Pharmaceutical Sciences. 2018;42(3):169-75.

55. Treur M, Baca E, Bobes J, Canas F, Salvador L, Gonzalez B, et al. The cost-effectiveness of paliperidone extended release in Spain. Journal of Medical Economics. 2012;15 Suppl 1:26-34.

56. Treur M, Heeg B, Moller HJ, Schmeding A, van Hout B. A pharmaco-economic analysis of patients with schizophrenia switching to generic risperidone involving a possible compliance loss. BMC Health Services Research. 2009;9:32.

57. Yang L, Li M, Tao LB, Zhang M, Nicholl MD, Dong P. Cost-effectiveness of long-acting risperidone injection versus alternative atypical antipsychotic agents in patients with schizophrenia in China. Value in Health. 2009;12 Suppl 3:S66-9.

58. Yang YK, Tarn YH, Wang TY, Liu CY, Laio YC, Chou YH, et al. Pharmacoeconomic evaluation of schizophrenia in Taiwan: model comparison of long-acting risperidone versus olanzapine versus depot haloperidol based on estimated costs. Psychiatry and Clinical Neurosciences. 2005;59(4):385-94.

59. Zhao J, Jiang K, Li Q, Zhang Y, Cheng Y, Lin Z, et al. Cost-effectiveness of olanzapine in the first-line treatment of schizophrenia in China. Journal of medical economics. 2019;22(5):439-46.

60. Zeidler J, Mahlich J, Greiner W, Heres S. Cost Effectiveness of Paliperidone Palmitate for the Treatment of Schizophrenia in Germany. Applied Health Economics and Health Policy. 2013;11(5):509-21.
